# Mobile App–Based Tracking of Daily Explicit and Implicit Sense of Agency Among Adults Living in Japan: Longitudinal Observational Study

**DOI:** 10.2196/77607

**Published:** 2026-08-05

**Authors:** Yoshiro Shirai, Eriko Sugisaki, Roberto Legaspi, Ryunosuke Togawa, Akihiro Sasaki, Akihiro Miyamoto, Motoyuki Sanada, Kae Nakajima, Nao Kobayashi, Yasushi Naruse

**Affiliations:** 1 Health and Medical Research Group, Think Tank Division, KDDI Research, Inc, 2-1-15 Ohara, Fujimino-shi, Saitama, 356-8502, Japan, 81 70-3509-0089; 2 Collaborative AI Group, AI Division, KDDI Research, Inc, Fujimino-shi, Saitama, Japan; 3 Center for Information and Neural Networks (CiNet), Advanced ICT Research Institute, National Institute of Information and Communications Technology, Kobe-shi, Hyogo, Japan

**Keywords:** sense of agency, general explicit SoA, local implicit SoA, mobile apps, digital health, interindividual variation, intraindividual variation, within-individual variation, between-individual variation, mobile phone, intentional binding, temporal binding

## Abstract

**Background:**

Sense of agency (SoA) is the subjective experience of controlling one’s actions and their effects in the world. SoA is important for behavioral change and goal achievement, and its measurement via digital devices has gained attention in health promotion and therapeutic interventions.

**Objective:**

This study describes longitudinal changes and individual variations in general explicit sense of agency (GenExp-SoA) and local implicit sense of agency (LocalImp-SoA), measured in daily life using a mobile app, and examines their associations with demographic and personality traits.

**Methods:**

One hundred adults in Japan participated in a 3-month, prospective observational survey, completing daily mobile app-based assessments of GenExp-SoA (using the Sense of Agency Scale) and LocalImp-SoA (using effect binding with the Libet clock method). Personality traits were measured via the Big Five questionnaire. Linear mixed-effects models were used to evaluate longitudinal changes and interactions with personal characteristics for both types of SoA and their relationship. We also examined state persistence and cross-lagged effects using continuous-time structural equation modeling. A total of 13,391 paired observations were analyzed.

**Results:**

GenExp-SoA decreased over time (β=−0.008, *P*<.001). The decrease was notable for women (β=−0.019, *P*<.001) and participants with extraversion scores higher than the median (β=−0.019, *P*<.001). An increase was observed in those aged 50‐59 years (β=0.023, *P*<.001). LocalImp-SoA also decreased (β=−0.003, *P*=.02), but its association with personal characteristics was limited. No significant association was found between the 2 forms of SoA. The variance patterns differed between measures, with a high intraclass correlation coefficient of 0.77 for GenExp-SoA and a very low intraclass correlation coefficient of 0.02 for LocalImp-SoA, indicating predominant within-individual variability for the latter. Continuous-time structural equation modeling showed stronger self-persistence for GenExp-SoA, minimal persistence for LocalImp-SoA, and negligible cross-lagged effects. Findings were robust in bootstrap and sensitivity analyses.

**Conclusions:**

The 2 forms of SoA measured in daily life settings displayed distinct temporal patterns, as well as different associations with individual characteristics. These findings may inform the design and evaluation of epidemiological studies and digital health interventions targeting the SoA, including measurement strategies. The results support lower-frequency, between-individual assessments for explicit agency and high-frequency, within-individual designs for implicit agency.

## Introduction

Sense of agency (SoA) is the subjective experience of having control over one’s own actions and influencing the external world through intentional effort [1]. The SoA is critical for distinguishing one’s actions from those of others [2]. It also functions as an internal reward, promoting movement selection and execution [3], and can influence decision-making related to behavioral change and goal achievement [4,5]. Multiple task-level and social context factors influence SoA, including the fluency of action selection and the extent of choice or coercion [6-13]. As many behavioral interventions manipulate these determinants, such as autonomy and choice support, feedback, and social collaboration, the SoA can function as a proximate, reactive indicator of mechanisms related to perceived control and engagement. Therefore, we consider the SoA as an indicator for evaluating the acceptability and effectiveness of interventions, as detailed below.

SoA is shaped by autonomy vs coercion, environmental regularities, and multisensory congruence and feedback. Discrepancies in these signals have been shown to reduce perceived control [14-16]. Neurocomputational models suggest that SoA is caused by hierarchical inference processes such as active inference and predictive coding and that disruptions in these processes are associated with neuropsychiatric and functional disorders [17]. Taken together, these accounts suggest 2 related but distinct layers: an explicit, reportable contextual appraisal shaped by autonomy or coercion, and an implicit multisensory sensorimotor process based on timing and sensory match. In this study, we frame SoA as a 2-layered construct in which explicit appraisals (integrating background information about the environment, internal knowledge about the world, and background beliefs) and implicit sensorimotor coupling shape behavior via motivational and affective routes [18-21]. Here, we used the Sense of Agency Scale (SoAS) to index general explicit sense of agency (a general sense of control, regardless of the situation; hereafter referred to as GenExp-SoA) [22,23] and outcome (temporal) binding in the Libet clock task as an indirect marker of local, implicit sensorimotor coupling (a momentary, task-dependent, nonconscious link between action and outcome; LocalImp-SoA hereafter) [18,24]. Hereafter, we refer to these as the 2 forms of the SoA. As these 2 measures target different layers of agency, they may show dissociable dynamics in free-living settings. Therefore, assessing both provides complementary information.

With the increasing use of digital devices such as smartphones in health promotion and therapeutic interventions [25,26], many digital therapeutic software programs and health care apps have been developed [27-29]. Additionally, the SoA can act as an inner psychological strength that protects individuals from the harmful effects of stress and adversity by helping to maintain motivation, purposeful action, and a sense of control [18,19]. Affective processing may also mediate the relationship between SoA and action regulation, influencing health behaviors [20]. In this context, SoA is a relevant construct that can be measured and potentially targeted through digital interventions. We assessed both layers to better capture how SoA changes in real-life digital contexts. Given this operationalization, modeling SoA dynamics can inform the timing and manner of support delivery. In particular, when the explicit agency is low or declining, interventions can shift toward autonomous support, and when it is high or increasing, task difficulty can increase gradually. When the implicit agency is unstable or declining, the task and notification load can be temporarily reduced, and the reliance on passive sensing can increase until stability returns. Frequent active assessments are burdensome in real-life settings; thus, passively sensed behavioral signals (eg, activity, phone use, heart rate variability [HRV], and location variability) can serve as low-burden proxies for tracking SoA dynamics between periodic reanchoring and direct assessments, and preliminary evidence indicates that they encode relevant aspects of SoA dynamics [30]. When validated rigorously and reanchored periodically to direct SoA assessments, such models could support just-in-time adaptive interventions that are contingent on agency [30].

Most previous studies on the SoA have been conducted in controlled laboratory environments that promote active engagement [31,32]. Therefore, to apply SoA in therapeutic interventions and health promotion using digital devices, it is necessary to measure SoA in daily life and examine its association with behavioral changes, psychological indicators, or health outcomes. In 2022, Legaspi et al [33] first reported SoA in daily life over a period longer than one month, using a mobile app during tasks such as meal recording. They reported that GenExp-SoA varied among individuals, increased over time, and strongly influenced the pursuit and achievement of specific goals, such as recording actions and achieving healthy eating [33]. They also reported no correlation between the 2 SoA measures, suggesting that their influence on outcomes may differ depending on individual psychological traits, highlighting the need to analyze their distinct changes and variances [33]. Furthermore, the SoA has been linked to health and well-being-related adjustment, thereby supporting motivation and goal pursuit [18-20]. This association underscores the importance of SoA in goal pursuit and achievement, which are critical not only for health but also for social behavior and education [11,13,14,33].

To advance research on this topic, evaluating the feasibility of daily assessments of the SoA and analyzing their changes over the long term at both the population and individual levels are necessary. Identifying differences in the SoA according to population background factors may also contribute to hypothesis generation in future epidemiological research. However, such detailed analyses remain limited, particularly under free-living conditions and are not driven by goal pursuit. Compared with the prior app-based study of SoA during goal pursuit (Legaspi et al [33]), this study focuses on free-living assessments without an assigned goal or prescribed goal-directed activities, and allows describing the baseline temporal properties of the 2 forms of the SoA in daily life. We extend prior research [33] by tracking both measures for 3 months in a larger and age- and sex-balanced adult sample while reporting key measurement properties for study design (eg, within- and between-individual variance and cross-lagged dynamics). Accordingly, our objectives were to characterize longitudinal changes and variation within and between individuals in both SoA measures, explore associations of individual characteristics and measurement timing with SoA trajectories over time, and examine the relationship between the 2 forms of SoA.

## Methods

### Study Participants

Participants were recruited via online research panels operated by third-party research companies in Japan. The target population included men and women aged 20 to 60 years living in Japan who were registered with these research companies and were invited as part of an on-site experiment measuring brain activity. All participants had previously completed an online preliminary survey, and recruitment was designed to balance gender and age distributions as much as possible. Individuals who met the exclusion criteria were not recruited (see Table S1 in Multimedia Appendix 1). All 120 individuals who consented to participate were informed about the study procedures and provided consent for the onsite survey. The analysis ultimately included 100 participants (mean age 44.0, SD 8.8 y) who consented to the daily life survey and for whom online preliminary survey data were available. Participants who did not participate in the daily life survey or who lacked online preliminary survey data were excluded. The daily life surveys were conducted between November 29, 2023, and April 15, 2024.

This exploratory study was not designed to test a specific effect size; therefore, no a priori sample size calculation was performed. We pragmatically sampled approximately 120 adults. To assess robustness, we conducted sensitivity analyses and internal validation via a cluster bootstrap at the participant level (see Methods, Internal Validity Assessment and Sensitivity Analyses).

### Online Preliminary Survey

An online screening survey was conducted before enrollment, and participants answered all 13 questions from the SoAS (SoAS-13) [22,23], the full set of items in this scale to measure GenExp-SoA, and questions on occupation, exercise habits (defined as exercising more than 1 h/wk), and Big Five personality traits (extraversion, agreeableness, conscientiousness, neuroticism, and openness) [34,35]. Age and gender information was obtained from the registration records of the third-party research companies.

### Mobile App-Based Survey of Daily Life

Participants received instructions before the survey and installed the custom-built app developed by our team on their smartphones. The app consisted of the following tasks, which were conducted in sequence as a single set of surveys: (1) the SoAS questionnaire, (2) the Libet clock task, (3) a visual analog scale for 7 emotions, (4) the cognitive task, and (5) Russell’s circumplex model plot. Despite collecting data from all parts, only the data from parts (1) and (2) were analyzed in this study. Data from parts (3) through (5) were obtained for other research purposes and were not included in the present analysis. The participants were asked to complete each survey daily as it was delivered to their smartphones for 3 months starting from a specified date. During the experimental period, the surveys were delivered randomly 3 times a day between waking and sleeping hours. The participants were asked to complete these surveys at least once a day within one hour of delivery. Participant compensation was based on the app-based survey recording rate, as described in the Ethical Considerations section.

### Assessment of GenExp-SoA

Although 13 questions from the SoAS were used in the preliminary survey, the number of items was reduced in the mobile app-based survey to minimize participant burden from completing tasks 3 times daily. Following Legaspi et al [33], three items assessing positive agency were selected and validated from the SoAS: (1) “I am in full control of what I do”; (2) “My behavior is planned by me from the very beginning to the very end”; and (3) “I am completely responsible for everything that results from my actions.” Participants responded to these items on the app using a 15-point visual analog scale ranging from “strongly agree” (15) to “strongly disagree” (1), with a default value of 8. The GenExp-SoA in this study was evaluated as the total score of these 3 questions per participant (range: 3‐45 points). The internal consistency of the 3 items was excellent, with a Cronbach α of 0.92 (95% CI 0.916 to 0.921) calculated across all the responses. The correlations of each item with the total score ranged from 0.90 to 0.95, indicating high reliability (Table S2 in Multimedia Appendix 1).

### Assessment of Local Implicit Sense of Agency

**Figure 1.**
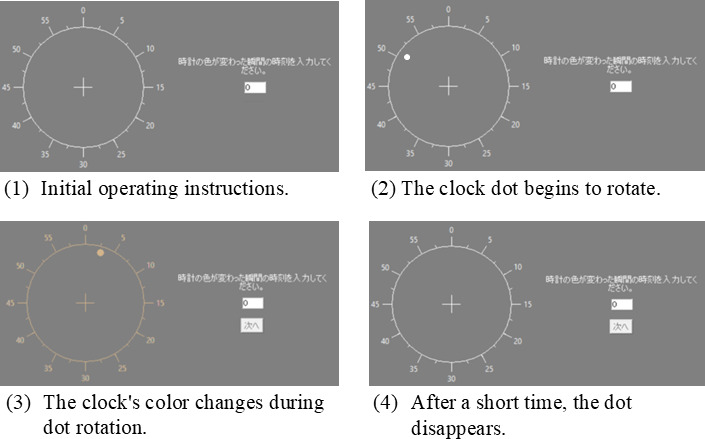
Two sensory tasks to measure local implicit SoA in the app. SoA: sense of agency.

In this study, the Libet clock displayed a clock-like dial on the app screen with 24 ticks and numbers every 2 ticks, along with a dot rotating clockwise in a 2560 ms cycle. The participants performed 2 sensory tasks using the Libet clock method: one without a behavioral task (baseline) and one with a task (operant). In these tasks, participants entered the time displayed at the moment the color of the Libet clock changed (Figure 1). Effect binding, calculated by subtracting the operant outcome judgment error from the baseline outcome judgment error, was used as the measure of LocalImp-SoA (range −30 to 30 points). The differences were calculated as signed shortest circular differences on the clock scale. Therefore, the following formula was used: (1) baseline outcome judgment error=baseline true time−baseline participant recorded value; (2) operant outcome judgment error=operant true time−operant participant recorded value; and (3) effect binding=baseline outcome judgment error−operant outcome judgment error. A positive effect binding indicates shortened time perception due to the behavioral task, suggesting an SoA, whereas a negative effect binding indicates decreased SoA.

Regarding baseline session, (1) first, participants were instructed to enter the time in numerical values when the clock’s color changed. (2) The dot began rotating without the participant’s operation. (3) The color of the clock changed during the rotation of the dot. (4) Participants recorded the time at the moment the clock color changed. Regarding operant session, (1) participants were instructed to click the start button to start the clock and then enter the numerical value when the clock color changed. (2) The dot began rotating immediately after the start button was clicked. (3) The color of the clock changed during the rotation of the dot. (4) The participants recorded the time at the moment the clock color changed. Thus, the major difference between the 2 sessions is whether the clock rotation begins without participant action (baseline session) or is triggered by participant action (operant session).

### Data Quality Control and Outlier Handling

To promote data quality and consistency, several procedures were implemented.

Task delivery times in the app were customized by each participant to align with their daily routines, thereby enhancing compliance and ecological validity. During data collection, the research team monitored the participants’ app usage and survey completion rates. The team provided technical support and reminders to participants who had markedly low response rates or experienced other issues. All data entries were automatically assigned timestamps to track response timing and identify irregularities. In the analytic dataset, 39 participants had observation windows (first to last completed day +1) longer than 90 days (maximum 116 d), reflecting missed days and occasional app reinstalls or resets. Days with ≥4 completed survey sets occurred in 535 of 6210 (8.62%) participant-day observations with at least one completed survey. These issues can occur in routine smartphone use and were treated as part of real-world use conditions. For the measurements of SoA, outliers were defined as observations exceeding ±3 SD from the pooled mean across all participants and days and were excluded. In total, 71 (0.5%) observations were excluded for GenExp-SoA, and 352 (2.5%) observations were excluded for LocalImp-SoA as outliers. The analytic dataset comprised 13,391 paired observations in which both measures were available, and all analyses used these paired records.

Missing data occurred across days and within days. No variables were completely missing across all time points. Missing values were not imputed.

### Statistical Analysis

#### Basic Characteristics of Study Participants

The mean and SD of age and each Big Five trait score, as well as the number and percentage by age group, gender, occupation category, and exercise habit category, were calculated for all participants and for quartiles of GenExp-SoA as measured in the online preliminary survey (ie, based on the SoAS score from the 13 questions). Their 95% CIs were also computed.

#### Measurements of the 2 Forms of SoA in Daily Life

The mean and SD were calculated for the entire period, as well as for each period (<30, 30‐59, and ≥60 d) for the following variables: number of task completion days, number of tasks completed per day, and daily average of each SoA measure. The period categories were defined using the integer day index (elapsed days from study start), which was also used in subsequent longitudinal analyses. In addition, the median and IQR were calculated for daily average baseline and operant outcome judgment errors, as well as the within-day and between-day variances of each SoA measure. Corresponding 95% CIs were computed for the mean estimates and for the medians.

#### Longitudinal Changes and Within- and Between-Individual Variance in the 2 Forms of SoA

We fitted linear mixed models (restricted maximum likelihood estimation) with nested random intercepts for participants and for days within participants and a fixed linear effect of time (elapsed days from the start of this study, coded as an integer day index). The objective of this random intercept specification was to provide a variance decomposition and an intraclass correlation coefficient (ICC). Analyses were conducted for all participants and stratified by gender, age group, Big Five trait scores (split into the median), day of the week, and time of day categories defined by the Japan Meteorological Agency (12 AM-5:59 AM, 6 AM-8:59 AM, 9 AM-2:59 PM, 3 PM-5:59 PM, 6 PM-11:59 PM). Time of day was modeled as a categorical variable based on the timestamp of survey completion. No random slopes were used in the variance decomposition models. We did not include random slopes for time at the participant level in the ICC models because, under a random slope specification, the between-individual variance (and therefore the ICC) becomes time-dependent. This contrasts with our goal of a single ICC summary describing measurement characteristics. The ICC was calculated as the variance at the participant level divided by the sum of variances at the participant, participant by day, and residual levels, conditional on fixed effects. To examine differences in longitudinal changes by participant characteristics, models with an interaction term between time and group were fitted. Specifically, we used time×group interaction models. The time slopes for each group were derived from the main time effect and the corresponding interaction term (eg, time+time:group).

#### Association Between the 2 Forms of SoA

We used random intercepts and random slopes specific to each participant for LocalImp-SoA, thereby allowing the within-individual association to vary across individuals. This model assessed contemporaneous associations within paired observations. The elapsed day index was not included as a covariate because longitudinal change and lagged dynamics were evaluated in separate longitudinal and dynamic models. The dependent variable was GenExp-SoA, the fixed effect was LocalImp-SoA, and the random effect was the participant. Estimates were obtained for a crude model and a model adjusted for age, sex, and each Big Five trait score. Big Five trait scores were entered in their original (not standardized) units. Therefore, coefficients for these covariates correspond to a 1-point increase in their respective scale.

Continuous-time structural equation modeling was used to estimate state changes over time for the 2 SoA measures. This approach enabled examination of state persistence and lagged effects between the explicit and implicit SoA measures. Bayesian estimation in Stan was used to sample from posterior distributions and summarize parameters at the population level. We fitted a continuous-time structural equation model using ctsem and transformed the fitted continuous-time parameters into discrete-time parameters at Δt=1 day for presentation and interpretation. The number of iterations for model fitting was set to 1500, with 4 chains for the Markov chain Monte Carlo method and a thinning interval of 2. Uncertainty is summarized using 95% credible intervals. Additionally, parameters based on elapsed days were visualized from the posterior distribution of the fitted model.

#### Internal Validity Assessment and Sensitivity Analyses

To assess the internal validity of our findings, we performed 1000 iterations of bootstrap resampling at the participant level for the primary longitudinal and association analyses of the 2 forms of SoA. For the continuous-time structural equation modeling, model validity and uncertainty were assessed using autocorrelation function plots of residuals and posterior predictive checks. The independence of parameters between the 2 forms of the SoA was evaluated via correlation among posterior parameter estimates.

Sensitivity analyses for the association between the 2 forms of SoA included (1) analyses using all available observations, (2) analyses with both random intercept and random slope models in the primary dataset, and (3) analyses in the primary dataset in which LocalImp-SoA was classified into 3 qualitative groups (<–0.5, –0.5 to 0.5, and ≥0.5), with further stratification by the median of the total values of baseline and operant outcome judgment errors to account for differences in task compliance. A generalized additive mixed model was used to test for nonlinear associations and slope heterogeneity among participants.

To assess potential nonlinearity in the longitudinal time trend, we also conducted a sensitivity analysis replacing the fixed linear effect of time with a natural spline term (ns (time, *df*=3)) in the primary linear mixed models for each SoA measure, while retaining the same random intercept structure (participants and days within participants); model comparisons were conducted using maximum likelihood.

#### Statistical Analysis Software

All statistical analyses were conducted using R (version 4.3.2; R Foundation) [36]. Linear mixed models were fitted using the ‘lmer’ function in the *‘lme4*' package [37]. Generalized additive (mixed) models were fitted with the *mgcv* package (using restricted maximum likelihood and bam with AR(1) correlation for daily series) [38,39]. Bootstrap resampling and model tidying were performed with the broom.mixed package [40]. Continuous-time structural equation modeling was specified using the ‘ctModel’ function and fitted using the ‘ctStanFit’ function, with parameter estimation from the posterior distribution using the ‘ctStanDiscretePars’ function in the *‘ctsem*’ package [41]. Multiplicity adjustments were not planned for exploratory analyses, and 2-sided *P* values were reported as informational values for type I errors.

### Ethical Considerations

This research received ethical approval from the Ethics Committee of the National Institute of Information and Communications Technology (N230192300). All study procedures involving human participants were conducted in accordance with the principles outlined in the Declaration of Helsinki and its subsequent revisions. Before participation, individuals received a detailed explanation of this study’s purpose and procedures, and written informed consent was obtained using documents approved by the ethics committee. All the participants voluntarily agreed to participate in this study after fully understanding the information provided.

Participant records were pseudonymized using hashed IDs, and the analytic dataset contained no direct personal identifiers. Data were stored and shared via access-controlled research servers with IP-based access restrictions and 2-factor authentication.

Participants received cash-equivalent points based on their app-based survey recording rate, according to a prespecified stepwise schedule approved by the ethics committee and explained before enrollment. Compensation ranged from JP ¥10,000 to JP ¥15,000 per participant (approximately US $67 to $100), with the minimum provided for a recording rate of ≤30% and the maximum for >90% to ≤100%.

## Results

### Basic Characteristics of Study Participants

Table 1 presents the basic characteristics of the participants, with the main values summarized. Compared with the lower quartiles (Q1 and Q2) of GenExp-SoA as measured using the 13-item SoAS, participants in the higher quartiles (Q3 and Q4) were older and had higher scores for extraversion, agreeableness, conscientiousness, and openness, with lower neuroticism. A greater proportion of males was observed in Q1. These are descriptive comparisons without statistical testing. The corresponding 95% CIs are reported in Table S3 in Multimedia Appendix 1.

**Table 1.** Basic characteristics according to quartiles of general explicit SoA^a^ measured by the full set of the SoAS^b^ at prebaseline. 95% CIs for each value are provided in Table S3 in Multimedia Appendix 1.

Variable	Q1 (n=24)	Q2 (n=24)	Q3 (n=28)	Q4 (n=24)	All (N=100)
Total SoAS score, mean (SD)	50.1 (2.1)	56.5 (2.8)	67.8 (4.6)	83.7 (4.9)	64.7 (13.1)
Age (years), mean (SD)	42.2 (9.9)	41.8 (7.7)	47.0 (8.4)	44.3 (8.7)	44.0 (8.8)
Age group (years), n (%)					
20-39	9 (37.5)	11 (45.8)	5 (17.9)	7 (29.2)	32 (32.0)
40‐49	9 (37.5)	11 (45.8)	11 (39.3)	10 (41.7)	41 (41.0)
50-59	6 (25.0)	2 (8.3)	12 (42.9)	7 (29.2)	27 (27.0)
Sex, n (%)					
Male	19 (79.2)	11 (45.8)	13 (46.4)	14 (58.3)	57 (57.0)
Female	5 (20.8)	13 (54.2)	15 (53.6)	10 (41.7)	43 (43.0)
Job category, n (%)					
Company employee	15 (62.5)	11 (45.8)	14 (50.0)	17 (70.8)	57 (57.0)
Public employee	3 (12.5)	2 (8.3)	1 (3.6)	1 (4.2)	7 (7.0)
Self-employed	2 (8.3)	1 (4.2)	1 (3.6)	1 (4.2)	5 (5.0)
Part-timer	1 (4.2)	3 (12.5)	5 (17.9)	2 (8.3)	11 (11.0)
Homemaker	1 (4.2)	2 (8.3)	2 (7.1)	2 (8.3)	7 (7.0)
Student	1 (4.2)	0 (0.0)	1 (3.6)	0 (0.0)	2 (2.0)
Unemployed	0 (0.0)	1 (4.2)	0 (0.0)	0 (0.0)	1 (1.0)
Other	1 (4.2)	4 (16.7)	4 (14.3)	1 (4.2)	10 (10.0)
Exercise habit of at least 1 hour/week, n (%)					
Yes	12 (50.0)	13 (54.2)	15 (53.6)	12 (50.0)	52 (52.0)
No	12 (50.0)	11 (45.8)	13 (46.4)	12 (50.0)	48 (48.0)
Big Five, mean (SD)					
Extraversion	14.3 (4.4)	13.8 (4.7)	15.4 (4.8)	16.5 (5.1)	15 (4.8)
Agreeableness	19.6 (3.5)	17.8 (4.5)	19.5 (3.9)	21.8 (5.1)	19.7 (4.4)
Conscientiousness	22.4 (3.9)	21.1 (5.4)	23.1 (5.7)	26.2 (6.1)	23.2 (5.6)
Neuroticism	15 (3.9)	16 (5.6)	14.5 (5.6)	11.8 (4.0)	14.3 (5.0)
Openness	17.9 (4.3)	15.1 (4.6)	17.3 (5.0)	19.3 (4.9)	17.4 (4.9)

a SoA: sense of agency.

b SoAS: Sense of Agency Scale.

### Measurements of the 2 Forms of SoA in Daily Life

Table 2 shows the measurement characteristics of the 2 forms of the SoA in daily life. We also report completion-based adherence metrics in Table 2, including the response day rate and the number of completed survey sets per observation day. The corresponding 95% CIs are provided in Table S4 in Multimedia Appendix 1. The average number of measurements per day increased in the latter half of the survey period, whereas the daily average GenExp-SoA decreased. The daily average baseline outcome judgment error and operant outcome judgment error decreased in the latter half of the survey period; however, no clear change was observed for LocalImp-SoA at the group level. The within-day variance in the daily average of the explicit measure was relatively stable across periods, whereas the between-day variance appeared higher in the latter half of the survey period. On the other hand, no clear change was observed in either the within-day or between-day variance for the implicit measure. To visualize measurement frequency over time, we present the mean number of completed survey sets per participant per day in Figure S3 in Multimedia Appendix 1.

**Table 2.** Measurements of general explicit and local implicit SoA by mobile apps in daily life. Observation period per participant (last date−first date+1): mean 78.2 (SD 18.5) days.

Variable	Day 1‐29	Day 30‐59	Day 60+	All period
Participants, n	100	98	90	100
Observations, n	4376	4654	4361	13,391
Measurement days, mean (SD)	22.0 (7.1)	21.2 (8.3)	20.3 (14.6)	61.0 (20.3)
Response day rate^a^	0.79 (0.24)	0.73 (0.28)	0.90 (0.18)	0.80 (0.22)
Completed survey sets per observation day^a^	1.53 (0.75)	1.59 (0.80)	1.96 (0.72)	1.70 (0.67)
Mean GenExp-^b^SoA^c^ /d, mean (SD)	33.1 (8.6)	32.7 (9.0)	31.5 (8.6)	32.5 (8.8)
Mean LocaIImp-^d^SoA /d, mean (SD)	0.06 (2.70)	0.06 (2.40)	−0.07 (2.13)	0.02 (2.44)
Mean baseline OJE^e^ /d, median (IQR)	3.0 (1.5‐6.2)	2.8 (1.5‐5.2)	2.4 (1.4‐4.0)	2.8 (1.5‐5.1)
Mean operant OJE /d, median (IQR)	3.2 (1.6‐6.0)	2.8 (1.4‐5.3)	2.5 (1.4‐4.1)	2.8 (1.4‐5.2)
Within day var^f^ of GenExp-SoA, median (IQR)	2.0 (0.5‐8.0)	2.0 (0.5‐6.3)	2.0 (0.3‐8.0)	2.0 (0.5‐8.0)
Within day var of LocalImp−SoA, median (IQR)	3.5 (0.9‐10.8)	3.2 (0.8‐9.0)	3.1 (0.8‐8.7)	3.2 (0.9‐9.3)
Between day var of mean GenExp-SoA/d, median (IQR)	8.0 (2.8‐19.9)	7.2 (2.7‐18.2)	12.6 (4.9‐24.2)	8.8 (2.8‐20.9)
Between day var of mean LocalImp−SoA/d, median (IQR)	3.2 (2.3‐6.0)	3.1 (2.1‐6.0)	3.3 (2.5‐4.5)	3.2 (2.3‐5.2)

a Adherence metrics were computed from timestamped completed survey sets only because the records of survey prompt delivery times were not available. Response day rate=(number of days with ≥1 completed survey set in the period)/(number of days in that period within each participant’s observation window). Completed survey sets per observation day=(number of completed survey sets in the period)/(number of days in that period within each participant’s observation window). Period-specific estimates were computed among participants whose observation periods covered each period.

b GenExp-: general explicit.

c SoA: sense of agency.

d LocalImp-: local implicit.

e OJE: outcome judgment error.

f var: variance.

### Changes in SoA Over Time and Differences Across Individuals and Measurement Times

Table 3 shows longitudinal changes and within-individual and between-individual variances in GenExp-SoA according to individual characteristics and measurement timing on the basis of the main mixed-effects model analysis. The fixed-effect coefficients for elapsed days represent the expected change per 1 day in the original scale (possible range: 3‐45 points). The bootstrap estimates and 95% CIs for internal validation are presented in Tables S5 and S6 in Multimedia Appendix 1. In the main analysis, elapsed days were associated with a decrease in GenExp-SoA (β=−0.008, *P*<.001), with more pronounced decreases among women (β=−0.019, *P*<.001) and among those with extraversion scores higher than the median (β=−0.019, *P*<.001), while those aged 50‐59 years showed an increase (β=0.023, *P*<.001). In the bootstrap analysis, these effect sizes were generally consistent, and the 95% CIs for women (β=−0.020, 95% CI −0.041 to 0.003) and for those aged 50‐59 years (β=0.024, 95% CI 0.007 to 0.039) were consistent with the main results. For the other subgroups, no substantial associations were observed. In a sensitivity analysis of the primary mixed-effects model, replacing the linear time term with a natural spline (ns (time, *df*=3)) under the same random intercept structure, the model fit did not improve (*P*=.80). Variance component estimates were similar between analyses. The within-individual variance was 20.2, which was greater on Sundays (24.2) and lower among those aged 50‐59 years (14.5) and during the time period of 12 AM to 5:59 AM (16.8). The between-individual variance was 66.3, which was higher among women (73.2) and those with extraversion scores below the median (83.0) and lower among those with openness scores higher than the median (49.1).

**Table 3.** Longitudinal changes and within- and between-individual variance in general explicit SoA^a^ by individual and timing characteristics. Statistical model: linear mixed model (restricted maximum likelihood). Fixed effect: linear time (elapsed days) and, where indicated, interactions between time and group; random effect: random intercepts for participants and for days nested within participants (no random slopes).

	n^b^	Times/d^c^	Fixed effect	*P* value	*P*-int^d^^e^	Within-var^f^	Between-var^g^	ICC^h^^i^
GenExp^j^-SoA	100	2.07 (0.49)	−0.008	<.001^k^	N/A^l^	20.2	66.3	0.77
Sex								
Male	57	2.04 (0.55)	0.002	.37	Ref^m^	19.9	58.3	0.75
Female	43	2.10 (0.41)	−0.019	<.001^k^	<.001^k^	20.5	73.2	0.78
Age (years)								
20‐39	32	2.20 (0.48)	−0.022	<.001^k^	Ref	20.8	64.7	0.76
40‐49	41	2.06 (0.48)	−0.009	.003^k^	.005^k^	22.8	67.6	0.75
50‐59	27	1.93 (0.50)	0.023	<.001^k^	<.001^k^	14.5	67.8	0.82
Big Five								
Ext^n^≤Md^o^	48	2.18 (0.47)	0.003	.34	Ref	18.7	83.0	0.82
Ext>Md	52	1.97 (0.50)	−0.019	<.001^k^	<.001^k^	21.7	51.4	0.70
Agr^p^≤Md	50	2.07 (0.52)	−0.010	<.001^k^	Ref	21.7	64.4	0.75
Agr>Md	50	2.07 (0.47)	−0.004	.16	.13	18.5	63.6	0.77
Con^q^≤Md	51	2.02 (0.58)	−0.013	<.001^k^	Ref	22.7	66.8	0.75
Con>Md	49	2.11 (0.39)	−0.001	.63	.004^k^	17.7	59.4	0.77
Neu^r^≤Md	56	1.98 (0.5)	−0.011	<.001^k^	Ref	19.1	69.5	0.78
Neu>Md	44	2.18 (0.47)	−0.004	.23	.06	21.4	63.5	0.75
Opn^s^≤Md	47	2.18 (0.53)	−0.008	.004^k^	Ref	19.1	76.6	0.80
Opn>Md	53	1.97 (0.44)	−0.007	.02^k^	.66	21.3	49.1	0.70
Day of the week								
Monday	1907	2.08 (0.56)	−0.008	.15	Ref	20.8	64.8	0.76
Tuesday	1897	2.12 (0.57)	−0.013	.03^k^	.73	20.3	64.7	0.76
Wednesday	1872	2.07 (0.59)	−0.008	.16	.93	19.3	64.7	0.77
Thursday	1979	2.12 (0.56)	0.001	.89	.45	17.9	66.2	0.79
Friday	1933	2.09 (0.58)	−0.006	.29	.86	20.0	66.0	0.77
Saturday	1849	1.99 (0.57)	−0.008	.15	.81	22.8	60.8	0.73
Sunday	1954	2.10 (0.50)	−0.014	.02^k^	.99	24.2	61.6	0.72
Time of day								
12 AM-5:59 AM	4532	1.23 (0.17)	−0.011	<.001^k^	Ref	16.8	65.8	0.80
6 AM‐8:59 AM	2167	1.07 (0.07)	−0.002	.65	.33	17.9	65.4	0.78
9 AM‐2:59 PM	4213	1.21 (0.16)	−0.008	.02^k^	.24	21.2	65.9	0.76
3 PM‐5:59 PM	400	1.02 (0.06)	0.022	.06	.003^k^	23.0	51.7	0.69
6 PM‐11:59 PM	2079	1.08 (0.14)	−0.007	.14	.66	19.2	60.0	0.76

a SoA: sense of agency.

b Number of participants for demographic and psychological variables; number of observations for day of the week and time of the day.

c Number of measurements per day per participant (mean [SD]).

d *P*-int: *P* value for interaction.

e *P* value for the time-by-group interaction term.

f Within-var: within-individual variance.

g Between-var: between-individual variance.

h ICC: intraclass correlation coefficient.

i Intraclass correlation coefficient=between-individual variance/total variance.

j GenExp-: general explicit.

k *P* values <.05.

l N/A: not applicable.

m Ref: reference.

n Ext: extraversion.

o Md: median.

p Agr: agreeableness.

q Con: conscientiousness.

r Neu: neuroticism.

s Opn: openness.

Similar analyses for LocalImp-SoA are summarized in Table 4. The fixed-effect coefficients for elapsed days represent the expected change per 1 day in the original scale (possible range −30 to 30). The bootstrap estimates and 95% CIs for internal validation are presented in Tables S7 and S8 in Multimedia Appendix 1. In the main analysis, elapsed days were associated with a decrease in LocalImp-SoA (β=−0.003, *P*<.05). Similarly, replacing the linear time term with a natural spline (ns (time, *df*=3)) did not improve model fit for LocalImp-SoA (*P*=.14). Among the Big Five traits, participants whose agreeableness scores were higher than the median showed a greater decrease (β=−0.005, *P*<.01). During the time period of 3:00 PM to 5:59 PM, a relatively large decrease was observed (β=−0.014, *P*=.06, *P*-interaction=.07). In the bootstrap analysis, these associations were generally consistent. The 95% CI for all participants (β=−0.003, 95% CI −0.005 to −0.0003), for women (β=−0.004, 95% CI −0.008 to −0.0005), and for those with agreeableness scores above the median (β=−0.005, 95% CI −0.009 to −0.002) were consistent with the main analysis. The other subgroups did not show notable associations. Variance component estimates showed similar patterns in both analyses. The within-individual variance was 8.7, which was greater among those aged 50‐59 years (12.2) and the time period 3:00 PM to 5:59 PM (12.7) and lower among those aged 20‐39 years (6.7). The between-individual variance of the implicit measure was 0.14.

**Table 4.** Longitudinal changes and within- and between-individual variance in local implicit SoA by individual and timing characteristics. Statistical model: linear mixed model (restricted maximum likelihood). Fixed effect: linear time (elapsed days) and, where indicated, interactions between time and group; random effect: random intercepts for participants and for days nested within participants (no random slopes).

	n^a^	Times/d^b^	Fixed effect	*P* value	*P*-int^c^^d^	Within-var^e^	Between-var^f^	ICC^g^^h^
LocalImp-^i^SoA^j^	100	2.07 (0.49)	−0.003	.02^k^	N/A^l^	8.7	0.14	0.02
Sex								
Male	57	2.04 (0.55)	−0.001	.40	Ref^m^	8.6	0.12	0.01
Female	43	2.10 (0.41)	−0.004	.008^k^	.17	8.9	0.17	0.02
Age (years)								
20‐39	32	2.20 (0.48)	−0.002	.16	Ref	6.7	0.13	0.02
40‐49	41	2.06 (0.48)	−0.003	.03	.57	8.4	0.09	0.01
50‐59	27	1.93 (0.50)	−0.001	.74	.69	12.2	0.24	0.02
Big Five								
Ext^n^≤Md^o^	48	2.18 (0.47)	−0.003	.049^k^	Ref	8.4	0.10	0.01
Ext>Md	52	1.97 (0.50)	−0.002	.18	.73	9.0	0.19	0.02
Agr^p^≤Md	50	2.07 (0.52)	−0.001	.69	Ref	8.0	0.15	0.02
Agr>Md	50	2.07 (0.47)	−0.005	.002^k^	.03	9.5	0.13	0.01
Con^q^≤Md	51	2.02 (0.58)	−0.002	.20	Ref	9.0	0.12	0.01
Con>Md	49	2.11 (0.39)	−0.003	.03^k^	.54	8.4	0.17	0.02
Neu^r^≤Md	56	1.98 (0.5)	−0.003	.049^k^	Ref	8.9	0.11	0.01
Neu>Md	44	2.18 (0.47)	−0.002	.18	.61	8.5	0.16	0.02
Opn^s^≤Md	47	2.18 (0.53)	−0.002	.14	Ref	7.9	0.10	0.01
Opn>Md	53	1.97 (0.44)	−0.003	.06	.67	9.5	0.18	0.02
Day of the week								
Monday	1907	2.08 (0.56)	−0.005	.08	Ref	8.9	0.01	0.00
Tuesday	1897	2.12 (0.57)	−0.002	.46	.41	9.0	0.10	0.01
Wednesday	1872	2.07 (0.59)	−0.003	.36	.54	9.1	0.17	0.02
Thursday	1979	2.12 (0.56)	−0.002	.37	.66	8.7	0.16	0.02
Friday	1933	2.09 (0.58)	−0.001	.60	.35	8.6	0.18	0.02
Saturday	1849	1.99 (0.57)	0.002	.48	.08	8.0	0.03	0.00
Sunday	1954	2.10 (0.50)	−0.003	.21	.62	8.9	0.27	0.03
Time of day								
12 AM-5:59 AM	4532	1.23 (0.17)	−0.001	.67	Ref	8.1	0.12	0.02
6 AM‐8:59 AM	2167	1.07 (0.07)	−0.004	.14	.30	8.6	0.08	0.01
9 AM‐2:59 PM	4213	1.21 (0.16)	−0.002	.22	.67	9.5	0.14	0.01
3 PM‐5:59 PM	400	1.02 (0.06)	−0.014	.06	.07	12.7	0.35	0.03
6 PM‐11:59 PM	2079	1.08 (0.14)	−0.002	.38	.66	7.9	0.07	0.01

a Number of participants for demographic and psychological variables; number of observations for the day of the week and time of the day.

b Number of measurements per day per participant (mean [SD]).

c *P*-int: *P* value for interaction.

d *P* value for the interaction between time and group.

e Within-var: within-individual variance.

f Between-var: between-individual variance.

g ICC: intraclass correlation coefficient.

h Intraclass correlation coefficient=between-individual variance/total variance.

i LocalImp-: local implicit.

j SoA: sense of agency.

k *P* values <.05.

l N/A: not applicable.

m Ref: reference.

n Ext: extraversion

o Md: median.

p Agr: agreeableness.

q Con: conscientiousness.

r Neu: neuroticism.

s Opn: openness.

### Association Between the 2 Forms of SoA

Table 5 shows the results of fitting LocalImp-SoA to GenExp-SoA using a linear mixed model. No significant associations were observed in either the crude model or the model adjusted for multiple variables. Bootstrap analysis also revealed no association between these parameters (Table S9 in Multimedia Appendix 1). In sensitivity analyses, no association was observed when all observations were used without outlier exclusion or when random intercept and random slope models were applied to the primary dataset. Similarly, no association was observed when the implicit measure was classified into 3 qualitative groups or when analyses were stratified by the median of the total value of baseline and operant outcome judgment errors (Table S10 in Multimedia Appendix 1). The generalized additive mixed model did not clearly support the average nonlinear association (Figure S1 in Multimedia Appendix 1). The slopes specific to each participant were better (ΔAIC [Akaike information criterion]=29), even though the average smoothing was not significantly curvilinear (*P*=.14). Comprehensive details are provided in Tables S11-S13 in Multimedia Appendix 1.

**Table 5.** Association between general explicit SoA^a^ and local implicit SoA. Objective variable: general explicit sense of agency. Statistical model: linear mixed-effects model using restricted maximum likelihood estimation. Fixed effect: local implicit SoA. Random effect: participant intercept and slope. The 95% CIs for fixed effects were calculated using the profile likelihood method.

	Fixed effect	SE	*P* value	95% CI
Crude model	0.011	0.016	.47	−0.020 to 0.043
Fully adjusted model^b^	0.006	0.034	.86	−0.056 to 0.073

a SoA: sense of agency.

b Adjusted for age, sex, and all Big Five personality traits.

**Figure 2.**
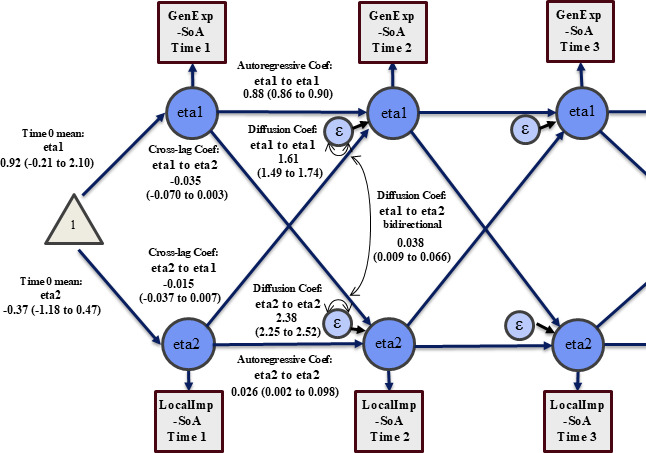
Population parameters estimated by continuous-time structural equation modeling for the 2 forms of the SoA. Squares represent observed variables, circles denote latent variables, triangles indicate the initial mean values of latent variables at time 0, and ε represents system noise affecting the latent variables. eta1 corresponds to GenExp-SoA, and eta2 corresponds to local implicit sense of agency (LocalImp-SoA). Parameters are shown in discrete time form implied by the continuous-time model (Δ*t*=1 day). The autoregressive and cross-lagged coefficients reflect persistence and cross-process prediction over a 1-day interval, and the diffusion coefficient represents the strength of random fluctuations or unpredictable external influences on the latent variables (system noise). Bayesian uncertainty is summarized using 95% credible intervals. coef: coefficient; eta1: explicit agency; eta2: implicit agency; GenExp-: general explicit; LocalImp-: local implicit; SoA: sense of agency.

The estimated population mean values of each parameter for the latent variables of GenExp-SoA (eta1) and LocalImp-SoA (eta2) obtained from continuous-time structural equation modeling are shown in Figure 2. The autoregressive coefficient of eta1 in discrete time was 0.88 (95% credible interval 0.86 to 0.90). For eta2, the persistence was minimal (0.03, 95% credible interval 0.002 to 0.098). The cross-lagged coefficients were −0.035 (eta1 to eta2; 95% credible interval −0.070 to 0.003) and −0.015 (eta2 to eta1; 95% credible interval −0.037 to 0.007). Both measures were strongly influenced by system noise (ie, unpredictable external influences and random variations), with diffusion coefficients of 1.61 (95% credible interval 1.49 to 1.74) and 2.38 (95% credible interval 2.25 to 2.52), respectively.

**Figure 3.**
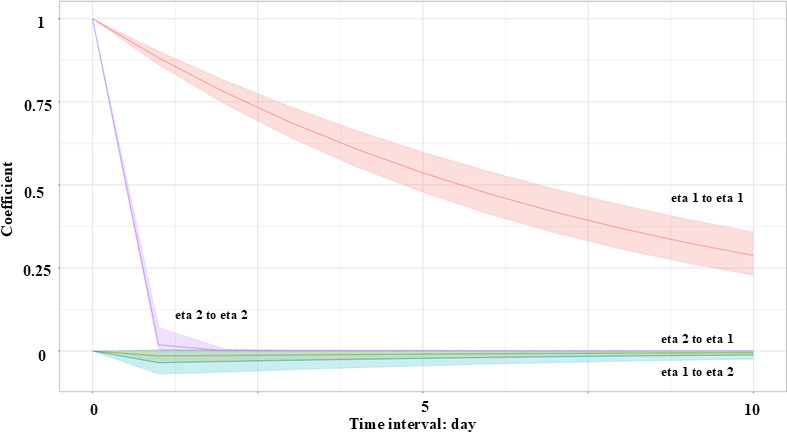
Temporal regressions for the independent standardized coefficients in the continuous-time structural equation model. This figure illustrates autoregressive and cross-lagged coefficients in discrete time for the 2 latent states. Eta1 corresponds to GenExp-SoA, and eta2 corresponds to LocalImp-SoA. The y-axis shows the coefficients with system noise standardized to 1.0 for visualization, and the x-axis indicates lag in days. Colored curves correspond to “eta1 to eta1,” “eta2 to eta2,” “eta1 to eta2,” and “eta2 to eta1,” with shaded bands indicating 95% credible intervals. eta1: explicit agency; eta2: implicit agency; GenExp-: general explicit; LocalImp-: local implicit; SoA: sense of agency.

The autoregressive and cross-lagged coefficients in discrete time as a function of elapsed days are shown in Figure 3, with the system noise standardized to 1.0 for visualization. The persistence of GenExp-SoA (eta1 to eta1) remained but gradually decreased over 10 days, whereas the very small persistence for LocalImp-SoA (eta2 to eta2) dissipated within approximately 2 days.

The estimated posterior correlation between the 2 SoA measures was –0.13 (95% credible interval –0.39 to 0.13), indicating uncertainty about the association (Table S14 in Multimedia Appendix 1). Summary statistics of the posterior distributions at the individual level for each period (<30, 30‐59, and ≥60 d) and each SoA are presented in Table S15 in Multimedia Appendix 1. The observed values mostly fell within the model’s predictive intervals, although these intervals were wide throughout this study (±18 for GenExp-SoA and ±5 for LocalImp-SoA). For GenExp-SoA, the estimated SD of the random effects (population SD 7.9) was similar to that of the observed data (SD 8.8), whereas for LocalImp-SoA, the estimated measurement error variance was unstable (mean 10.3, SD 25.8). Both SoA measures exhibited large diffusion coefficients, suggesting that the wide predictive intervals were attributable mainly to the large data variance, individual differences, and high levels of noise. The residual autocorrelation plots revealed no significant autocorrelation, suggesting that the model adequately captured the temporal structure (Figure S2 in Multimedia Appendix 1). However, the width of the credible intervals remained relatively large (approximately ±0.05 to ±0.1).

## Discussion

### Principal Findings

This current study tracked the 2 forms of SoA over 3 months in the daily life of 100 participants, using a longer duration and larger sample size than a previous study [33], which had a duration of approximately one month with fewer than 50 participants. In contrast to the previous study conducted under conditions involving goal pursuits, our study assessed the SoA without direct experimental manipulation, allowing observation under conditions closer to daily life. We observed small declines in the overall mean across all participants, stronger between- than within-individual variance, and high daily persistence for GenExp-SoA, but high within-individual variance and minimal persistence for LocalImp-SoA. No clear association was observed between the 2 measures in linear, nonlinear, and lagged effect analyses. Sensitivity analyses and continuous-time structural equation modeling supported these patterns.

These results inform hypothesis generation, study design, estimation of sample sizes, and other considerations in population science targeting the SoA and may guide strategies to evaluate the SoA in digital health interventions. Our findings support a 2-layer account in naturalistic settings. In free-living settings without an assigned goal or prescribed goal-pursuit activities, unmeasured day-to-day changes in attention and context may contribute to fluctuations in sensorimotor coupling (ie, eta2), which may partly explain the observed dissociation from reflective self-appraisals. Eta1 is slower and more stable, while eta2 is more context-dependent and more noise-sensitive.

### Within- and Between-Individual Variation in the 2 Forms of SoA

The within- and between-individual variations in the 2 SoA measures differed in this study. GenExp-SoA, which has a high ICC, appears to adequately evaluate cross-sectional between-individual differences. In contrast, LocalImp-SoA, which has a very low ICC, suggests that the within-individual variation is very large, which may make it challenging to evaluate cross-sectional between-individual differences (Tables 3 and 4). The continuous-time structural equation modeling results support this difference. GenExp-SoA exhibited substantial autoregressive persistence, with a slower decay over time (high daily persistence; attenuation over ≈10 d), whereas LocalImp-SoA showed near-zero persistence (attenuation within ≈2 d), greater diffusion, and wide posterior predictive intervals, as well as a near-zero posterior correlation with the explicit measure (Figures 2 and 3, Tables S14 and S15 in Multimedia Appendix 1). In the continuous-time structural equation modeling, the 2 latent states were anchored by the observed measures of eta1 and eta2. The eta1 likely reflects a slowly varying explicit appraisal of control grounded in general agency beliefs, with daily adjustments influenced by mood, motivation, and contextual predictability. The eta2 likely indexes sensorimotor coupling between actions and outcomes at a short time-scale; is sensitive to attentional load, fatigue, adherence, and task valence and freedom of choice; and includes notable system noise. Accordingly, under free-living conditions, we expected the 2 measures to show dissociable dynamics, with eta2 being more contextually labile (ie, sensitive to contextual variation) than explicit metacognitive appraisals. Consequently, GenExp-SoA may be used to compare between-individual differences even with lower-frequency assessment, whereas LocalImp-SoA is better captured through higher-frequency, within-individual designs in free-living settings.

These patterns are also consistent with hierarchical computational accounts of SoA in predictive coding and active inference [17,21]. In brief, these accounts describe SoA as arising from hierarchical inference with 2 coupled levels: higher-level reflective beliefs about control and lower-level sensorimotor predictions (and prediction errors) that link actions to outcomes. In these accounts, GenExp-SoA, which is based on reflective self-reports, may reflect more stable metacognitive appraisals and higher-level beliefs about control, consistent with its high ICC and strong persistence. By contrast, LocalImp-SoA, indexed by outcome (temporal) binding, may depend more strongly on lower-level sensorimotor inference and short time-scale fluctuations that are sensitive to contextual variability, attentional state, fatigue, and uncertainty in temporal judgments, consistent with its low ICC, minimal persistence, and large diffusion. The near-zero association between the 2 measures may suggest that, in free-living settings, each measure is influenced by partly distinct components and measurement noise, even if the underlying levels are coupled in principle. This interpretation is tentative because prediction errors and relevant contextual or physiological covariates were not directly measured. Nevertheless, it provides a concise link between the observed temporal dynamics and computational accounts of agency.

Although LocalImp-SoA showed large diffusion and wide predictive intervals, these unstable observations were not only due to system noise. They may also reflect genuine short-time-scale variance in implicit sensorimotor coupling in mobile environments. Accordingly, improving measurement stability in mobile, free-living settings will likely require increasing the number of trials per session and measuring and compensating for latencies specific to the device and the operating system. Temporal binding is associated with dispositional traits such as self-transcendence [42], and LocalImp-SoA likely reflects a dynamic sensorimotor process with psychosocial relevance. Therefore, it is preferable to assess this with a multimethod battery rather than a single proxy. Analytically, nonlinear and time-varying models may better capture real-life changes and test just-in-time adaptations triggered by momentary states in free-living settings [43-46], with SoA as a candidate trigger to be validated.

### Changes in SoA

In this study, a decline in the mean of each SoA measure across the cohort of participants was observed (Tables 3 and 4). Although the average change in both SoAs among participants was small, SoA can plausibly have cumulative implications for motivation and behavioral regulation because affective processing partially mediates the pathway from agency to action regulation [20], and agency functions as a dynamic psychological resource in health-related meaning-making and adjustment [19]. From a population-science perspective, even modest shifts in the cohort mean may still be relevant because they indicate a systematic group-level change rather than isolated individual fluctuations. Moreover, cohort-level averages can mask heterogeneity in individual trajectories and context-dependent fluctuations. This is consistent with the observed variance structure (high ICC for GenExp-SoA and low ICC for LocalImp-SoA) and the minimal persistence and large diffusion observed for LocalImp-SoA. In line with these observations, the decrease in GenExp-SoA reflects changes in participants’ awareness, whereas the decline in LocalImp-SoA indicates lower persistence, which is consistent with labile implicit coupling. Previous studies have reported associations between SoA and health, psychological, social, and environmental factors [14-16,47-49]. Thus, the observed decline in SoA may be attributable to unmeasured factors related to health, psychology, the environment, and social status. Such complex contextual changes may act at both the population and individual levels.

The observed decline in the SoA may also reflect seasonality. We note that environmental and seasonal covariates (eg, weather, illness seasonality, and work cycles) were not directly measured in this study. To our knowledge, no studies have reported seasonal variations in SoA. However, seasonal variations have been reported for indicators of health, psychological state, environmental factors, and social status, which are associated with the SoA [50-56], many of which have been linked in prior studies to processes that are relevant to agency [18,47-49]. In this study period (late fall to spring), SoA decreased on average. Previous studies have reported increases in weight [51], the prevalence of metabolic syndrome [50], and possible increases in depression in winter [52], as well as poorer scores on neuropsychological test batteries, such as visual and verbal attention, working memory, verbal ability, verbal fluency, and executive functioning [54]. Such seasonal changes across multiple factors may have contributed to the observed decline in the SoA and to differences in individual characteristics in this study. The contribution of seasonality may be nontrivial and it cannot be disentangled without explicitly modeling environmental covariates (eg, weather, illness seasonality, and work cycles). More broadly, seasonality may covary with reductions in multisensory congruence, constraints on autonomy, or disruptions of environmental regularities. These conditions can diminish perceived control and performance [14-18]. Taken together, these seasonality-related interpretations are speculative and hypothesis-generating.

In contrast to the results of the present study, Legaspi et al [33] reported an increase in GenExp-SoA from November 20, 2021, to December 31, 2021. Both studies measured GenExp-SoA using a mobile app in daily life. However, their observation window was late fall to early winter, whereas our observations spanned late fall to spring. In addition, the study by Legaspi et al [33] instructed participants to perform goal pursuit tasks. This contrast supports the idea that mastery experiences, autonomy, and structured feedback can increase eta1. Future studies should test this by explicitly manipulating and modeling these contextual factors in real-life settings [14,15,18].

### Interaction Between Time and Individual Characteristics for SoA

For GenExp-SoA, a slight increase was observed among men, whereas a decrease was observed among women (Table 3). With respect to personality, women are known to have higher levels of agreeableness and neuroticism [57,58]. In the present study, women had higher scores on neuroticism than men did, but the agreeableness scores were similar (data not shown); participants scoring above the median on these traits showed a smaller decrease in the explicit measures than those scoring below the median did (Table 3). These findings suggest that social, environmental, and biological factors related to sex may be associated with perceptions and self-disclosure of the SoA and that higher agreeableness and neuroticism may attenuate these decreases.

With respect to age groups, GenExp-SoA decreased in the 20‐39 age group, slightly decreased in the 40‐49 age group, and increased in the 50‐59 age group (Table 3). A prior large longitudinal study reported that extraversion, conscientiousness, and openness decrease across adulthood [59]. In our study, the older age groups had higher mean scores for extraversion, agreeableness, and conscientiousness but lower scores for neuroticism (data not shown). The GenExp-SoA decreased less among groups with high agreeableness, conscientiousness, and neuroticism scores than among those with low scores. By contrast, participants with high extraversion showed a greater decrease (Table 3). Reorganization of roles, socioeconomic demands, and regulatory styles, all linked to age, has the potential to influence SoA trajectories through motivational, cognitive, and affective routes [18-20,47]. The existence of known interrelations among sex, age, and personality suggests the necessity of conducting mediation and moderation tests based on a priori hypotheses. These assessments should include affective processing as a potential mediator [20]. In addition, the contextual moderators described in the Introduction should be considered, including autonomy vs coercion and environmental regularities [14-16].

### Association Between the 2 Forms of SoA

Although no linear, nonlinear, or cross-lagged associations were observed between the 2 SoA measures (Table 5, Figure S1 in Multimedia Appendix 1, and Figure 2), the nonlinear model suggested a slight increase in GenExp-SoA at high LocalImp-SoA, but this was not statistically significant and requires replication using larger, more diverse samples. This observation is consistent with the results of previous studies, demonstrating they are independent components in both experimental [60] and daily environments [33]. Existing theories suggest that eta1 reflects conscious self-control, whereas eta2 is related to unconscious behavioral control [48]. This dissociation is consistent with the distinct construct coverage of the 2 indicators. The SoAS depends on general agency beliefs that are independent of context, whereas outcome binding reflects local coupling between actions and effects that are specific to the task [18,22,24]. These measures are therefore complementary rather than interchangeable, and combining them provides a more comprehensive perspective. Prereflective states can inform explicit appraisals, and explicit appraisals can modulate prereflective coupling. Both associations are context-dependent and noise-sensitive. Several studies have indicated that eta2, as indexed by binding, is sensitive to outcome valence and the degree of choice freedom [8,15,61].

### Practical Implications for Digital Health and Behavioral Interventions

In digital health, eta1 can function as a tailoring variable for intervention delivery [62]. In the event of a downturn, interventions can accentuate recent successes, divide tasks into more manageable components, and provide precise and timely feedback [62,63]. These actions can support motivation, behavioral regulation, and adherence [18,20,62,63]. These findings also have implications for AI-driven adaptive digital interventions that tailor the timing and content of support to fluctuations in eta1 and eta2 in real time. In all the analyzed models of this study, no association was found between the 2 forms of SoA. However, modeling slopes that are specific to each participant improved the model fit, highlighting the importance of within-individual patterns (Tables S11 and S12 in Multimedia Appendix 1). This is a hypothesis-generating proposition: instability in LocalImp-SoA may be a marker of acute cognitive load or fatigue when considered together with a brief vigilance test or HRV. This is consistent with evidence that brief mobile vigilance tests and wearable HRV are sensitive indicators of acute fatigue and stress in applied and real-world settings [64,65]. In such instances, timely adaptation can postpone demanding inputs and rely more on passive sensing. This approach has the potential to promote sustained engagement [62,63]. However, to our knowledge, no study has jointly measured intentional and temporal binding with HRV and vigilance testing, which warrants further research. The passive sensing of behavioral data, including heart rate, activity, smartphone and app use, and GPS, can be integrated with brief active probes to estimate changes in the SoA and to trigger support in real time that is contingent on agency [30]. However, these models must undergo rigorous internal and external validation, periodic reanchoring to ground-truth SoA assessments, and fairness checks before deployment.

### Strengths and Limitations

This study is the first to detail voluntary measurements of the 2 forms of SoA over 3 months using smartphones in free-living environments, with a sample balanced for age and sex, a larger sample size, and a longer observation period than prior mobile SoA research. This study combines multilevel and continuous-time modeling with internal validation.

However, there are several limitations. First, this study has a descriptive, exploratory, and observational design. Therefore, causal inference is constrained, and observed associations may include unmeasured confounding, including unmodeled environmental and seasonal covariates such as weather, illness seasonality, and work cycles. Second, because data collection using mobile apps depended on participants’ smartphone usage habits, environmental conditions, and adherence, it is not possible to exclude self-selection bias, self-report bias, behavioral bias, and environmental bias with network factors. In addition, because participant compensation was linked to the app-based survey recording rate, compensation may have influenced input behavior, including recording frequency, continuation, and response accuracy. As compensation was not contingent on the content or values of SoA responses, it was unlikely to directly incentivize particular SoA reports. Additionally, device and operating system heterogeneity, occasional app reinstallations, and connectivity issues may have introduced nonclassical measurement error, meaning state-dependent or device-dependent errors (ie, systematic, nonrandom errors) and technical disruptions that may lead to skipped days or irregular completion patterns. However, these factors are inherent to routine real-world app deployment. Accordingly, evaluating the SoA under such constraints provides real-life valid and implementation-relevant estimates for digital health. Third, generalizability is restricted to adults aged 20‐60 years living in Japan, and it may not extend to other age groups or to patients with serious mental illness or sensory impairment. In addition, transposability to settings outside Japan, including those with different languages, health systems, or cultural contexts, remains unknown. Fourth, LocalImp-SoA exhibited large within-individual variability. Trial counts at the session level may have been insufficient, and using outcome judgment errors as a compliance proxy is imperfect. The collective consideration of these limitations indicates a direction for improvement. In addition, the 3-month high-frequency mobile assessment with internal validation provides reliable empirical and practical information for future SoA research and digital health applications.

### Future Directions

Future studies could integrate affective measures for mediation, contextual moderators such as autonomy and coercion or environmental regularities, and multimethod implicit batteries. They could also extend to diverse populations while leveraging computational accounts to guide feedback design aligned with intended and sensed outcomes. These steps may help clarify mechanisms, improve construct validity and generalizability, and inform the timing and content of adaptive interventions.

### Conclusions

This study used a mobile app to measure the 2 forms of SoA in daily life over 3 months, without specific tasks or goals. The temporal dynamics of the SoA were characterized, as well as their relationships with individual characteristics. GenExp-SoA showed greater between-individual than within-individual variation, whereas LocalImp-SoA showed very high within-individual variation. The mean explicit measures decreased over time across all participants, with greater declines observed among women and participants with high extraversion scores, while the decline was attenuated among participants with high conscientiousness scores. Conversely, GenExp-SoA tended to increase among participants aged 50‐59 years. The mean implicit measures also showed a slight decrease, but associations with individual characteristics were limited, and no association was observed between the 2 forms of SoA. These findings suggest that epidemiological studies and digital health interventions should account for the distinct measurement properties of the 2 forms of SoA, use lower frequency assessments for eta1, use high frequency designs at the individual level for eta2, and consider individual characteristics when interpreting SoA dynamics.

## Supplementary material

10.2196/77607 Multimedia Appendix 1 Figures and tables with additional study details.

## Abbreviations

AIC: Akaike information criterion
GenExp-SoA: general explicit sense of agency
HRV: heart rate variability
ICC: intraclass correlation coefficient
LocalImp-SoA: local implicit sense of agency
SoA: sense of agency
SoAS: Sense of Agency Scale
