# Supplementary material for: Mobile App–Based Tracking of Daily Explicit and Implicit Sense of Agency Among Adults Living in Japan: Longitudinal Observational Study
Source: JMIR Form Res. 2026 Aug 5;10:e77607. doi: 10.2196/77607 (PMC13446172; doi:10.2196/77607)
Supplement: Supplementary file 1 [file formative-v10-e77607-s001.pdf]

Table of Contents

Table S1: Conditions for exclusion from recruiting eligibility........ 3

Table S2: Reliability statistics for the three general explicit Sense of Agency items selected from the Sense of Agency Scale........ 4

Table S3: 95% confidence intervals for means and proportions of basic characteristics by quartiles of general explicit SoA at pre-baseline........ 5

Table S4: 95% confidence intervals for mean and median estimates of general explicit and local implicit SoA measured by a mobile app in daily life........ 6

Table S5: Bootstrap estimates of fixed and interaction effects in general explicit SoA by individual and timing characteristics........ 7

Table S6: Bootstrap estimates of variance components and ICC in general explicit SoA by individual and timing characteristics........ 8

Table S7: Bootstrap estimates of fixed and interaction effects in local implicit SoA by individual and timing characteristics........ 9

Table S8: Bootstrap estimates of variance components and ICC in local implicit SoA by individual and timing characteristics........ 10

Table S9: Bootstrap estimates of association between general explicit SoA and local implicit SoA........ 11

Table S10: Sensitivity analysis for association between general explicit SoA and local implicit SoA........ 12

Figure S1: Average smooth of implicit SoA predicting explicit SoA........ 14

Table S11: Generalized additive (mixed) model for the association between implicit and explicit SoA........ 15

Table S12: Comparison of AR(1)-adjusted GAMM with and without participant-specific slopes for implicit SoA by AIC.. 16

Table S13: k-index check for the implicit SoA smooth term.. 17

Table S14: Posterior correlations among parameter estimates in continuous-time structural equation modeling for explicit and implicit sense of agency.. 18

Table S15: Posterior predictive checks from continuous-time structural equation modeling for explicit and implicit sense of agency by study period.. 19

Figure S2: Residual autocorrelation from continuous-time structural equation modeling for explicit and implicit sense of agency.. 20

Figure S3: Residual autocorrelation from continuous-time structural equation modeling for explicit and implicit sense of agency.. 21

Table S1. Conditions for exclusion from recruiting eligibility

No.	Condition
1	Inability to use an Android device (OS version 10 or higher)
2	Left-handedness
3	Use of a pacemaker
4	History of brain surgery
5	Previous use of electrical nerve stimulators
6	History of neuropsychiatric disorders
7	Use of a hearing aid or ear prosthesis
8	Severe visual or hearing impairment
9	Regular use of glasses or contact lenses with astigmatism correction
10	Regular use of glasses or contact lenses and inability to see objects within 1 to 2 meters without them
11	Recent visits to an eye clinic due to symptoms such as distorted, blurred, or difficult-to-focus vision

Note: Items 9–11 were applied only to participants recruited in 2023.

Table S2. Reliability statistics for the three general explicit Sense of Agency items selected from the Sense of Agency Scale.

Statistic	Value
Cronbach's alpha	0.919
95% confidence interval (Feldt's method)	0.916–0.921

Item [a]	Mean	SD	Item-total correlation [b]	Alpha if item dropped [c]
Q1	10.7	3.19	0.95	0.85
Q2	10.5	3.30	0.93	0.88
Q3	11.3	2.95	0.90	0.91

Number of observations: 13,391

[a] Item statements:

Q1: I am in full control of what I do.

Q2: My behavior is planned by me from the very beginning to the very end.

Q3: I am completely responsible for everything that results from my actions.

Response scale: 15-point visual analog scale ranging from "strongly agree" to "strongly disagree".

[b] Pearson's correlation between each item and the sum of the other items.

[c] Cronbach's alpha if the item is removed from the scale.

Table S3. 95% confidence intervals for means and proportions of basic characteristics by quartiles of general explicit SoA at pre-baseline

Variable	Q1 (n=24)	Q2 (n=24)	Q3 (n=28)	Q4 (n=24)	All (n=100)
**Total SoAS score**	49.3 to 51.0	55.3 to 57.7	66.0 to 69.5	81.6 to 85.8	62.1 to 67.3
**Age**	38.0 to 46.3	38.6 to 45.1	43.8 to 50.3	40.7 to 48.0	42.2 to 45.7
**Age group, n (%)**					
≤ 39	21.2 to 57.3	27.9 to 64.9	7.9 to 35.6	14.9 to 49.2	23.7 to 41.7
40–49	21.2 to 57.3	27.9 to 64.9	23.6 to 57.6	24.5 to 61.2	31.9 to 50.8
≥ 50	12.0 to 44.9	2.3 to 25.8	26.5 to 60.9	14.9 to 49.2	19.3 to 36.4
**Sex, n (%)**					
Male	59.5 to 90.8	27.9 to 64.9	29.5 to 64.2	38.8 to 75.5	47.2 to 66.3
Female	9.2 to 40.5	35.1 to 72.1	35.8 to 70.5	24.5 to 61.2	33.7 to 52.8
**Job category, n (%)**					
Company employee	42.7 to 78.8	27.9 to 64.9	32.6 to 67.4	50.8 to 85.1	47.2 to 66.3
Public employee	4.3 to 31.0	2.3 to 25.8	0.6 to 17.7	0.7 to 20.2	3.4 to 13.7
Self-employed	2.3 to 25.8	0.7 to 20.2	0.6 to 17.7	0.7 to 20.2	2.2 to 11.2
Part-timer	0.7 to 20.2	4.3 to 31.0	7.9 to 35.6	2.3 to 25.8	6.3 to 18.6
Homemaker	0.7 to 20.2	2.3 to 25.8	2.0 to 22.6	2.3 to 25.8	3.4 to 13.7
Student	0.7 to 20.2	–	0.6 to 17.7	–	0.6 to 7.0
Unemployed	–	0.7 to 20.2	–	–	0.2 to 5.4
Other	0.7 to 20.2	6.7 to 35.9	5.7 to 31.5	0.7 to 20.2	5.5 to 17.4
**Exercise habit ≥1 h/week, n (%)**					
Yes	31.4 to 68.6	35.1 to 72.1	35.8 to 70.5	31.4 to 68.6	42.3 to 61.5
No	31.4 to 68.6	27.9 to 64.9	29.5 to 64.2	31.4 to 68.6	38.5 to 57.7
**Big Five**					
Extraversion	12.5 to 16.2	11.9 to 15.8	13.5 to 17.2	14.3 to 18.7	14.1 to 16.0
Agreeableness	18.1 to 21.0	15.9 to 19.6	18.0 to 21.0	19.7 to 23.9	18.8 to 20.5
Conscientiousness	20.7 to 24.0	18.8 to 23.4	20.9 to 25.3	23.6 to 28.7	22.1 to 24.3
Neuroticism	13.4 to 16.6	13.7 to 18.4	12.3 to 16.7	10.1 to 13.5	13.3 to 15.3
Openness	16.1 to 19.7	13.1 to 17.0	15.4 to 19.2	17.3 to 21.4	16.4 to 18.4

Abbreviations: SoA, sense of agency; SoAS, the sense of agency scale.
"–" indicates not applicable data.

Table S4. 95% confidence intervals for mean and median estimates of general explicit and local implicit SoA measured by a mobile app in daily life

Variable	Day 1–29	Day 30–59	Day 60–	All period
	Value			
Measurement days[a]	20.5 to 23.4	19.5 to 22.8	17.2 to 23.3	56.9 to 65.0
Measurement times/d[a]	1.9 to 2.0	2.2 to 2.3	2.3 to 2.4	2.2 to 2.2
Responded day rate[c]	0.74 to 0.83	0.67 to 0.78	0.86 to 0.94	0.76 to 0.84
Completed survey sets per observation day[c]	1.38 to 1.68	1.43 to 1.75	1.81 to 2.11	1.57 to 1.83
Mean GenExp-SoA /d[a]	32.8 to 33.5	32.3 to 33.1	31.2 to 31.9	32.3 to 32.7
Mean LocImp-SoA /d[a]	0.1 to 0.2	0.0 to 0.2	−0.2 to 0.0	0.0 to 0.1
Mean baseline OJE /d[b]	2.9 to 3.1	2.7 to 3.0	2.3 to 2.5	2.7 to 2.8
Mean operant OJE /d[b]	3.0 to 3.4	2.7 to 3.0	2.4 to 2.6	2.7 to 2.9
Within day var. of GenExp-SoA[b]	2.0 to 2.0	2.0 to 2.0	2.0 to 2.3	2.0 to 2.0
Within day var. of LocImp–SoA[b]	3.1 to 4.0	2.9 to 3.5	2.8 to 3.6	3.1 to 3.5
Between day var. of mean GenExp-SoA/d[b]	7.2 to 8.4	6.4 to 8.0	11.6 to 12.9	8.7 to 9.6
Between day var. of mean LocImp–SoA/d[b]	3.2 to 3.6	3.0 to 3.2	3.2 to 3.6	3.2 to 3.3

[a] 95% confidence interval for mean.
[b] 95% confidence interval for median.
Mean (SD) investigation period: 78.4 (18.4) days.

Abbreviations: GenExp-, general explicit; LocImp-, local implicit; OJE, outcome judgment error; SoA, sense of agency; var., variance.

Table S5. Bootstrap estimates of fixed and interaction effects in general explicit SoA by individual and timing characteristics

	Mean[a]	SD[a]	Lower[a]	Upper[a]	*P* value[b]	*P* for interaction[b]	Nboot[c]
**All**	-0.008	0.006	-0.020	0.005	.20	—	988
**Sex**							
Male	0.002	0.006	-0.009	0.014	.64	.08	988
Female	0.020	0.012	-0.041	0.003	.09		
**Age**							
20-39 years	-0.025	0.014	-0.049	0.004	.11	*ref*	988
40-49 years	-0.009	0.008	-0.024	0.008	.27	.35	
50-59 years	0.024	0.008	0.007	0.039	.006	.002	
**Big five**							
Ext ≤ median	0.002	0.006	-0.009	0.015	.72	*ref*	993
Ext > median	-0.020	0.011	-0.039	0.003	.11	.09	
Agr ≤ median	-0.011	0.008	-0.025	0.005	.14	*ref*	990
Agr > median	-0.005	0.011	-0.025	0.017	.64	.70	
Con ≤ median	-0.014	0.011	-0.033	0.008	.22	*ref*	992
Con > median	-0.002	0.006	-0.014	0.011	.77	.28	
Neu ≤ median	-0.012	0.009	-0.029	0.006	.25	*ref*	989
Neu > median	-0.004	0.009	-0.020	0.014	.66	.52	
Opn ≤ median	-0.007	0.007	-0.022	0.007	.29	*ref*	995
Opn > median	-0.009	0.011	-0.028	0.012	.40	.84	
**Day of the week**							
Monday	-0.009	0.007	-0.022	0.006	.25	*ref*	986
Tuesday	-0.011	0.007	-0.023	0.002	.10	.56	
Wednesday	-0.009	0.007	-0.022	0.005	.23	.94	
Thursday	-0.003	0.006	-0.014	0.009	.64	.17	
Friday	-0.011	0.007	-0.024	0.004	.16	.66	
Saturday	-0.007	0.007	-0.020	0.008	.32	.67	
Sunday	-0.009	0.008	-0.023	0.006	.23	.90	
**Time of the day**							
0:00–5:59	-0.010	0.007	-0.023	0.005	.19	*ref*	987
6:00–8: 59	-0.009	0.007	-0.023	0.005	.22	.80	
9:00–14: 59	-0.006	0.006	-0.018	0.006	.34	.36	
15:00–17: 59	0.004	0.013	-0.021	0.028	.72	.19	
18:00–23: 59	-0.013	0.007	-0.026	0.002	.08	.65	

Bootstrap resampling was performed at the participant (cluster) level with 1000 iterations. Mixed-effects models were fitted in each bootstrap sample.

[a] Mean: bootstrap mean; SD: bootstrap standard deviation; Lower and Upper: 2.5th and 97.5th percentiles of the bootstrap distribution.

[b] Bootstrap p values were calculated as twice the smaller proportion of bootstrap estimates above or below zero (two-sided test).

[c] The number in the "Nboot" indicates the number of bootstrap samples for which the model successfully converged according to the convergence criterion.

Abbreviations: Agr, Agreeableness; Con, Conscientiousness; Ext, Extraversion; Neu, Neuroticism; Opn, Openness; SoA, sense of agency.

Table S6. Bootstrap estimates of variance components and ICC in general explicit SoA by individual and timing characteristics

	Within-individual variance				Beween-individual variance				ICC[b]				Nboot[c]
	Mean[a]	SD[a]	Lower[a]	Upper[a]	Mean	SD	Lower	Upper	Mean	SD	Lower	Upper	
**All**	20.9	1.9	17.2	24.5	66.2	6.9	52.3	79.3	0.76	0.03	0.70	0.81	988
**Sex**													
Male	20.5	2.4	16.1	25.4	58.2	7.1	44.6	72.3	0.74	0.03	0.67	0.80	996
Female	21.1	2.6	16.3	26.1	73.2	12.1	47.6	94.9	0.77	0.04	0.68	0.84	992
**Age**													
20-39 years	21.8	3.4	15.0	28.1	64.0	15.1	37.8	88.1	0.74	0.06	0.61	0.84	994
40-49 years	23.3	2.9	17.7	29.3	67.5	10.7	45.4	87.8	0.74	0.04	0.65	0.81	991
50-59 years	15.6	3.0	10.1	20.8	67.7	9.5	48.4	84.8	0.81	0.04	0.73	0.88	997
**Big five**													
Ext ≤ median	19.3	2.6	14.2	24.7	82.3	12.2	58.6	105.5	0.81	0.03	0.73	0.86	994
Ext > median	22.1	2.5	17.3	26.8	51.5	5.6	40.4	62.3	0.70	0.04	0.63	0.76	995
Agr ≤ median	22.2	2.6	17.1	27.0	64.1	7.8	48.9	78.8	0.74	0.03	0.67	0.80	995
Agr > median	19.4	2.8	14.0	24.9	63.8	12.7	36.5	84.4	0.76	0.05	0.65	0.84	996
Con ≤ median	23.5	2.7	18.4	28.6	66.7	10.4	46.4	85.6	0.74	0.04	0.64	0.81	995
Con > median	18.1	2.5	13.3	23.1	59.2	9.7	38.4	75.0	0.76	0.04	0.67	0.83	995
Neu ≤ median	19.9	2.5	15.1	25.1	69.4	11.1	46.0	88.6	0.77	0.04	0.68	0.84	996
Neu > median	21.9	2.5	17.3	27.3	63.1	7.5	47.8	76.8	0.74	0.03	0.67	0.80	997
Opn ≤ median	19.8	2.4	15.3	24.3	76.7	11.0	53.5	97.2	0.79	0.03	0.72	0.85	993
Opn > median	21.9	2.7	16.5	27.0	48.9	5.4	38.1	59.4	0.69	0.04	0.61	0.76	988
**Day of the week**													
Monday	21.9	2.1	17.8	25.9	64.3	7.1	49.9	77.5	0.74	0.03	0.68	0.80	1000
Tuesday	20.8	2.3	16.3	25.1	64.3	6.5	51.2	76.5	0.75	0.03	0.69	0.81	999
Wednesday	20.1	2.1	16.1	24.1	64.3	6.9	50.5	76.4	0.76	0.03	0.70	0.81	997
Thursday	18.9	2.1	14.9	23.0	65.7	7.0	51.1	78.2	0.77	0.03	0.71	0.83	997
Friday	20.7	2.0	16.9	24.7	65.7	7.3	51.0	78.2	0.76	0.03	0.70	0.81	1000
Saturday	23.2	2.0	19.2	27.1	61.3	6.9	47.5	74.1	0.72	0.03	0.66	0.78	998
Sunday	24.8	2.4	20.5	29.6	61.5	7.0	47.5	74.6	0.71	0.03	0.64	0.77	999
**Time of the day**													
0:00–5:59	17.3	1.4	14.7	20.1	65.9	6.9	52.5	78.3	0.79	0.02	0.74	0.83	999
6:00–8: 59	18.1	1.5	15.3	21.0	65.1	7.2	50.0	78.3	0.78	0.03	0.72	0.82	999
9:00–14: 59	21.0	2.2	16.6	25.3	66.3	7.2	52.4	79.7	0.76	0.03	0.70	0.81	997
15:00–17: 59	23.4	6.5	12.3	34.6	51.5	8.8	34.0	68.6	0.69	0.08	0.53	0.84	998
18:00–23: 59	19.6	1.9	15.9	23.4	60.2	6.2	48.2	72.1	0.75	0.03	0.69	0.80	995

Bootstrap resampling was performed at the participant (cluster) level with 1000 iterations.

Mixed-effects models were fitted in each bootstrap sample.

[a] mean: bootstrap mean; sd: bootstrap standard deviation; lower and upper: 2.5th and 97.5th percentiles of the bootstrap distribution.

[b] Intraclass Correlation Coefficient = Between-individual variance / Total variance.

[c] The number in the "Nboot" indicates the number of bootstrap samples for which the model successfully converged according to the convergence criterion.

Abbreviations: Agr, Agreeableness; Con, Conscientiousness; Ext, Extraversion; ICC, Intraclass Correlation Coefficient; Neu, Neuroticism; Opn, Openness; SoA, sense of agency.

Table S7. Bootstrap estimates of fixed and interaction effects in local implicit SoA by individual and timing characteristics

	Mean[a]	SD[a]	Lower[a]	Upper[a]	*P* value[b]	*P* for interaction[b]	Nboot[c]
**All**	-0.003	0.001	-0.005	0.000	.03	—	980
**Sex**							
Male	-0.002	0.002	-0.005	0.002	.35	*ref*	990
Female	-0.004	0.002	-0.008	0.000	.03	.30	
**Age**							
20-39 years	-0.002	0.002	-0.006	0.001	.24	*ref*	982
40-49 years	-0.004	0.002	-0.008	0.000	.04	.50	
50-59 years	-0.001	0.003	-0.007	0.006	.82	.66	
**Big five**							
Ext ≤ median	-0.003	0.002	-0.007	0.001	.09	*ref*	983
Ext > median	-0.003	0.002	-0.006	0.001	.13	.93	
Agr ≤ median	-0.001	0.002	-0.004	0.003	.70	*ref*	983
Agr > median	-0.005	0.002	-0.009	-0.002	<.001	.05	
Con ≤ median	-0.002	0.002	-0.006	0.001	.18	*ref*	977
Con > median	-0.003	0.002	-0.007	0.000	.05	.64	
Neu ≤ median	-0.004	0.002	-0.008	0.000	.08	*ref*	982
Neu > median	-0.002	0.002	-0.005	0.001	.25	.44	
Opn ≤ median	-0.002	0.002	-0.005	0.001	.19	*ref*	985
Opn > median	-0.004	0.002	-0.008	0.000	.06	.55	
**Day of the week**							
Monday	-0.006	0.002	-0.011	-0.001	.02	*ref*	979
Tuesday	-0.003	0.002	-0.007	0.002	.21	.31	
Wednesday	-0.003	0.002	-0.008	0.001	.12	.39	
Thursday	-0.003	0.003	-0.008	0.001	.18	.41	
Friday	-0.002	0.002	-0.006	0.003	.49	.15	
Saturday	0.001	0.002	-0.004	0.006	.65	.02	
Sunday	-0.004	0.002	-0.008	0.000	.07	.47	
**Time of the day**							
0:00–5:59	-0.001	0.002	-0.005	0.002	.41	*ref*	987
6:00–8: 59	-0.006	0.003	-0.012	0.000	.04	.17	
9:00–14: 59	-0.002	0.002	-0.005	0.002	.33	.92	
15:00–17: 59	-0.014	0.007	-0.027	0.000	.05	.10	
18:00–23: 59	-0.003	0.003	-0.009	0.002	.23	.51	

Bootstrap resampling was performed at the participant (cluster) level with 1000 iterations.

Mixed-effects models were fitted in each bootstrap sample.

[a] Mean: bootstrap mean; SD: bootstrap standard deviation; Lower and Upper: 2.5th and 97.5th percentiles of the bootstrap distribution.

[b] Bootstrap p values were calculated as twice the smaller proportion of bootstrap estimates above or below zero (two-sided test).

[c] The number in the "Nboot" indicates the number of bootstrap samples for which the model successfully converged according to the convergence criterion.

Abbreviations: Agr, Agreeableness; Con, Conscientiousness; Ext, Extraversion; Neu, Neuroticism; Opn, Openness; SoA, sense of agency.

Table S8. Bootstrap estimates of variance components and ICC in local implicit SoA by individual and timing characteristics

	Within-individual variance				Beween-individual variance				ICC[b]				Nboot[c]
	Mean[a]	SD[a]	Lower[a]	Upper[a]	Mean	SD	Lower	Upper	Mean	SD	Lower	Upper	
**All**	8.9	0.6	7.8	10.1	0.1	0.0	0.1	0.2	0.01	0.003	0.009	0.02	980
**Sex**													
Male	8.7	0.9	7.1	10.5	0.1	0.0	0.0	0.2	0.01	0.005	0.004	0.02	995
Female	9.0	1.0	7.3	11.1	0.2	0.0	0.1	0.2	0.02	0.005	0.009	0.03	991
**Age**													
20-39 years	6.7	0.8	5.2	8.4	0.1	0.0	0.0	0.2	0.02	0.007	0.006	0.03	994
40-49 years	8.7	0.8	7.3	10.3	0.1	0.0	0.0	0.1	0.01	0.004	0.002	0.02	988
50-59 years	12.4	1.9	9.3	16.3	0.2	0.1	0.1	0.3	0.02	0.007	0.005	0.03	990
**Big five**													
Ext ≤ median	8.6	0.8	7.2	10.1	0.1	0.0	0.0	0.2	0.01	0.004	0.003	0.02	996
Ext > median	9.2	1.0	7.3	11.3	0.2	0.0	0.1	0.2	0.02	0.005	0.009	0.03	985
Agr ≤ median	8.1	0.7	6.7	9.6	0.1	0.0	0.1	0.2	0.02	0.006	0.007	0.03	985
Agr > median	9.7	1.0	7.9	11.8	0.1	0.0	0.1	0.2	0.01	0.003	0.006	0.02	991
Con ≤ median	9.2	0.9	7.5	11.1	0.1	0.0	0.0	0.2	0.01	0.004	0.003	0.02	985
Con > median	8.6	0.9	6.8	10.3	0.2	0.0	0.1	0.2	0.02	0.005	0.009	0.03	985
Neu ≤ median	9.1	1.0	7.4	11.3	0.1	0.0	0.0	0.2	0.01	0.004	0.004	0.02	991
Neu > median	8.7	0.8	7.2	10.3	0.1	0.0	0.1	0.2	0.02	0.005	0.007	0.03	986
Opn ≤ median	8.0	0.6	6.7	9.2	0.1	0.0	0.0	0.2	0.01	0.004	0.004	0.02	984
Opn > median	9.8	1.1	7.8	12.1	0.2	0.0	0.1	0.2	0.02	0.005	0.008	0.03	988
**Day of the week**													
Monday	9.1	0.7	7.8	10.6	0.0	0.0	0.0	0.1	0.00	0.005	0.000	0.02	990
Tuesday	9.2	0.8	7.8	10.9	0.1	0.1	0.0	0.3	0.01	0.010	0.000	0.03	989
Wednesday	9.1	0.8	7.6	10.7	0.2	0.1	0.0	0.4	0.02	0.012	0.000	0.04	997
Thursday	8.8	0.7	7.5	10.2	0.2	0.1	0.0	0.4	0.02	0.010	0.002	0.04	998
Friday	8.6	0.6	7.4	9.9	0.2	0.1	0.0	0.5	0.03	0.014	0.001	0.06	998
Saturday	8.1	0.7	6.8	9.6	0.1	0.1	0.0	0.2	0.01	0.007	0.000	0.02	990
Sunday	9.0	0.7	7.7	10.3	0.2	0.1	0.1	0.4	0.02	0.010	0.006	0.05	995
**Time of the day**													
0:00–5:59	8.4	0.6	7.3	9.6	0.1	0.0	0.0	0.2	0.01	0.005	0.005	0.02	985
6:00–8: 59	8.6	0.6	7.4	9.8	0.1	1.4	0.0	0.2	0.01	0.027	0.000	0.02	941
9:00–14: 59	9.7	0.8	8.1	11.1	0.1	0.0	0.1	0.2	0.01	0.005	0.005	0.02	982
15:00–17: 59	12.4	1.9	8.6	15.8	0.4	0.2	0.0	0.7	0.03	0.015	0.000	0.06	987
18:00–23: 59	8.2	0.6	7.1	9.2	0.1	0.0	0.0	0.2	0.01	0.005	0.000	0.02	984

Bootstrap resampling was performed at the participant (cluster) level with 1000 iterations. Mixed-effects models were fitted in each bootstrap sample.

[a] mean: bootstrap mean; sd: bootstrap standard deviation; lower and upper: 2.5th and 97.5th percentiles of the bootstrap distribution.

[b] Intraclass Correlation Coefficient = Between-individual variance / Total variance.

[c] The number in the "Nboot" indicates the number of bootstrap samples for which the model successfully converged according to the convergence criterion.

Abbreviations: Agr, Agreeableness; Con, Conscientiousness; Ext, Extraversion; ICC, Intraclass Correlation Coefficient; Neu, Neuroticism; Opn, Openness; SoA, sense of agency.

Table S9. Bootstrap estimates of association between general explicit SoA and local implicit SoA

Crude model	Fixed effect	SE	*P* value[a]	95% CI	Nboot[b]
Mean[c]	0.004	0.043	.63	-0.082 to 0.090	729
SD[c]	0.027	0.006	.25	0.034 to 0.025	
Lower[c]	-0.048	0.029	.11	-0.142 to 0.039	
Upper[c]	0.057	0.053	.99	-0.014 to 0.136	
**Fully adjusted model[d]**					
Mean	0.004	0.043	.63	-0.082 to 0.09	699
SD	0.027	0.006	.25	0.034 to 0.025	
Lower	-0.048	0.029	.11	-0.142 to 0.039	
Upper	0.057	0.053	.99	-0.014 to 0.136	

Bootstrap resampling was performed at the participant (cluster) level with 1000 iterations. Mixed-effects models were fitted in each bootstrap sample. Fixed effect: Local implicit SoA; Random intercept and random slope: participant.

[a] Bootstrap p values were calculated as twice the smaller proportion of bootstrap estimates above or below zero (two-sided test).
[b] The number in the "Nboot" indicates the number of bootstrap samples for which the model successfully converged according to the convergence criterion.
[c] Mean: bootstrap mean; SD: bootstrap standard deviation; Lower and Upper: 2.5th and 97.5th percentiles of the bootstrap distribution.
[d] Adjusted for age, sex, and all Big Five personality traits.

Table S10. Sensitivity analysis for association between general explicit SoA and local implicit SoA

Group	Fixed effect[a]	SE	*P* value	95% CI
**Crude model**				
All available observations[b]	0.010	0.031	.75	−0.051 to 0.071
Random intercept model	0.011	0.016	.47	−0.019 to 0.042
Random slope model	−0.097	0.101	.34	−0.298 to 0.105
Sum of judgment error < median[c]	0.019	0.049	.70	−0.078 to 0.116
Sum of judgment error ≥ median[c]	0.002	0.038	.96	−0.073 to 0.077
Total observations[d]				
LocalImp-SoA ≤ -0.5			**(ref)**	
LocalImp-SoA -0.5 to 0.5	−0.041	0.145	.38	−0.326 to 0.247
LocalImp-SoA ≥ 0.5	0.056	0.125	.32	−0.192 to 0.302
Sum of judgment error < median[c]				
LocalImp-SoA ≤ -0.5			**(ref)**	
LocalImp-SoA -0.5 to 0.5	0.045	0.156	.78	−0.261 to 0.361
LocalImp-SoA ≥ 0.5	0.091	0.172	.60	−0.247 to 0.434
Sum of judgment error ≥ median[c]				
LocalImp-SoA ≤ -0.5			**(ref)**	
LocalImp-SoA -0.5 to 0.5	−0.091	0.197	.65	−0.482 to 0.300
LocalImp-SoA ≥ 0.5	0.036	0.151	.81	−0.266 to 0.335
**Fully adjusted model[e]**				
All available observations	0.010	0.031	.76	−0.052 to 0.071
Random intercept model	0.011	0.016	.47	−0.019 to 0.042
Random slope model	−0.110	0.094	.25	−0.296 to 0.077
Sum of judgment error < median	0.017	0.049	.73	−0.080 to 0.114
Sum of judgment error ≥ median	0.002	0.038	.95	−0.073 to 0.077
Total observations				
LocalImp-SoA ≤ -0.5			**(ref)**	
LocalImp-SoA -0.5 to 0.5	−0.049	0.145	.74	−0.334 to 0.238
LocalImp-SoA ≥ 0.5	0.052	0.125	.68	−0.196 to 0.298
Sum of judgment error < median				
LocalImp-SoA ≤ -0.5			**(ref)**	
LocalImp-SoA -0.5 to 0.5	0.015	0.153	.92	−0.292 to 0.322
LocalImp-SoA ≥ 0.5	0.076	0.171	.66	−0.263 to 0.417
Sum of judgment error ≥ median				
LocalImp-SoA ≤ -0.5			**(ref)**	
LocalImp-SoA -0.5 to 0.5	−0.092	0.197	.64	−0.482 to 0.298
LocalImp-SoA ≥ 0.5	0.034	0.152	.82	−0.269 to 0.332

Objective variable: General explicit sense of agency (SoA).

Statistical model: Linear mixed-effects model using restricted maximum likelihood estimation.

[a] Fixed effect: Local implicit SoA; random intercept and random slope for each participant.

[b] Number of all available observations = 13,813.

[c] Median of sum of judgment error per day: 5.31. Number of observations; sum of judgment error < median = 6,648; sum of judgment error ≥ median = 6,743.

[d] Number of observations: Total = 13,391; for local implicit SoA categories: ≤ -0.5 = 4,818; -0.5 to 0.5 = 3,629; ≥ 0.5 = 4,944.

[e] Adjusted for age, sex, and all Big Five personality traits.

Abbreviations: SoA, sense of agency; SE, standard error; CI, confidence interval; LocalImp-, local implicit.

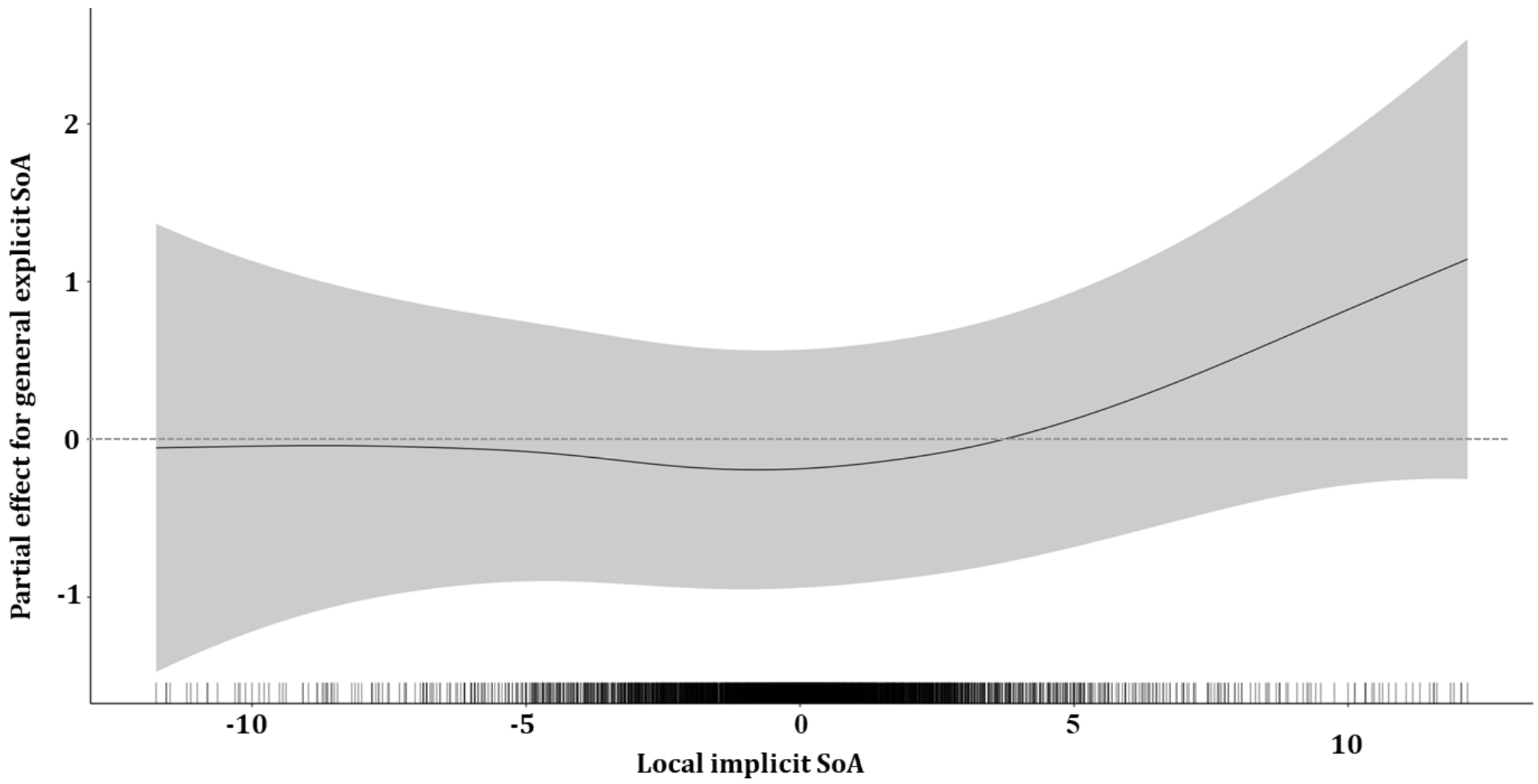

Figure S1: Average smooth of implicit SoA predicting explicit SoA

Abbreviations: AR(1), first-order autoregressive; GAM, generalized additive model; GAMM, generalized additive mixed model; SoA, Sense of Agency.

Objective variable: general explicit SoA.

Random effects via re-smooths: participant intercept and slope.

Predictor of interest: local implicit SoA (smooth term), adjusted for age, sex, and all Big Five traits

AR(1) rho was set to 0.37 (median participant-level lag-1 residual autocorrelation from the preliminary GAM). Average smooth of implicit SoA predicting explicit SoA, estimated with an AR(1)-adjusted generalized additive mixed model at daily resolution.

The solid line indicates the partial effect (centered smooth), and the shaded bands represent the 95% confidence intervals. Tick marks on the x-axis indicate the marginal distribution of daily implicit SoA.

Table S11. Generalized additive (mixed) model for the association between implicit and explicit SoA

Model	GAM	GAMM
**Method**	REML	fREML
**AR(1) autocorrelation**[a]	NA	0.37
**Smooth term:**		
s(LocalImp-SoA) edf	2.58	2.69
s(LocalImp-SoA) *P* value	.28	.14
s(Participant) edf	91.4	91.1
s(Participant) *P* value[b]	<.001	.98
s(LocalImp-SoA: Participant) edf	46.1	33.9
s(LocalImp-SoA: Participant) *P* value[b]	.003	.99
**Deviance explained (%)**	78.1	77.7
**Adjusted R2**	0.776	0.772
**AIC**	35450.9	34065.8

Abbreviations: AIC, Akaike information criterion; AR(1), first-order autoregressive; edf, Effective degrees of freedom; fREML, fast restricted maximum likelihood; GAM, generalized additive model; GAMM, generalized additive mixed model; LocalImp-SoA, Local implicit Sense of Agency.
Family/link: Gaussian/identity
Objective variable: general explicit SoA.
Random effects via re-smooths: participant intercept and slope.
Predictor of interest: local implicit SoA (smooth term), adjusted for age, sex, and all Big Five traits
[a] AR(1) rho was set to 0.37 (median participant-level lag-1 residual autocorrelation from the preliminary GAM).
[b] For re-smooths, *P* values are not inferential.

Table S12. Comparison of AR(1)-adjusted GAMM with and without participant-specific slopes for implicit SoA by AIC

Model (GAMM)	Random intercept and slope	Random intercept only
AIC	34065.9	34094.9
ΔAIC[a]	ref	29.0
Residual variance (σ2)[b]	15.8	15.9

Abbreviations: AIC, Akaike information criterion; AR(1), first-order autoregressive; REML, restricted maximum likelihood; GAM, generalized additive model; GAMM, generalized additive mixed model.
Family/link: Gaussian/identity
Objective variable: general explicit SoA.
Random effects via re-smooths: participant intercept (and slope where indicated)
Predictor of interest: local implicit SoA (smooth term), adjusted for age, sex, and all Big Five traits.
AR(1) rho was set to 0.370 (median participant-level lag-1 residual autocorrelation from a preliminary GAM).
Notes:
[a] The ΔAIC of 29.0 strongly indicates the importance of including participant specific slopes.
[b] The exclusion of slopes (random intercept only model) resulted in an increase in the residual variance.

Table S13. k-index check for the implicit SoA smooth term

Model	AR(1) autocorrelation[a]	k' (maximum basis dimension)	k-index	*P* value
GAM	Not Applicable	19	1.02	.86
GAMM	0.37	19	0.99	.34

Abbreviations: AR(1), first-order autoregressive; fREML, fast restricted maximum likelihood; GAM, generalized additive model; GAMM, generalized additive mixed model; SoA, Sense of Agency.
Family/link: Gaussian/identity.
Objective variable: general explicit sense of agency.
Random effects via re-smooths: participant intercept and slope.
Predictor of interest: local implicit SoA (smooth term), adjusted for age, sex, and all Big Five traits
[a] AR(1) rho was set to 0.370 (median participant-level lag-1 residual autocorrelation from the preliminary GAM).
Note: k index near 1 and a nonsignificant test indicate no undersmoothing. *P* value of very low with k index < 1 may suggest increasing k or addressing residual structure.

Table S14. Posterior correlations among parameter estimates in continuous-time structural equation modeling for explicit and implicit sense of agency

	Estimates mean	Estimates SD	95% credible interval
Posterior correlation	−0.13	0.13	−0.392 to 0.126

Posterior correlation among parameter estimates from the fitted continuous-time structural equation modeling.

Table S15. Posterior predictive checks from continuous-time structural equation modeling for explicit and implicit sense of agency by study period

Predicted mean	Predicted SD	Predicted 2.5th percentile	Predicted 97.5th percentile
**General explicit sense of agency**			
	**All period**		
32.8 (0.4); 32.1 to 33.7	9.1 (0.2); 8.8 to 9.5	15.1 (0.7); 14.0 to 16.3	50.5 (0.7); 49.2 to 51.7
	**Days 1-29**		
33.0 (0.4); 32.3 to 33.8	9.2 (0.2); 8.8 to 9.6	15.1 (0.7); 13.9 to 16.6	50.8 (0.8); 49.4 to 52.3
	**Days 30-59**		
32.7 (0.4); 32.0 to 33.6	9.1 (0.2); 8.7 to 9.5	15.1 (0.8); 13.7 to 16.8	50.3 (0.7); 48.9 to 51.5
	**Days ≥60**		
32.6 (0.4); 32.1 to 33.3	9.0 (0.3); 8.5 to 9.5	15.1 (0.8); 13.7 to 16.7	50.3 (0.8); 48.7 to 51.7
**Local implicit sense of agency**			
	**All period**		
0.023 (0.03); −0.03 to 0.07	2.4 (0.04); 2.4 to 2.6	−4.7 (0.1); −5.1 to −4.6	4.8 (0.1); 4.7 to 5.1
	**Days 1-29**		
0.006 (0.04); −0.11 to 0.07	2.5 (0.07); 2.4 to 2.7	−4.8 (0.2); −5.3 to −4.7	4.9 (0.1); 4.7 to 5.2
	**Days 30-59**		
0.036 (0.03); −0.01 to 0.09	2.4 (0.02); 2.4 to 2.4	−4.7 (0.1); −4.8 to −4.5	4.7 (0.1); 4.6 to 4.8
	**Days ≥60**		
0.036 (0.04); −0.06 to 0.12	2.4 (0.03); 2.4 to 2.5	−4.6 (0.1); −4.8 to −4.5	4.7 (0.1); 4.5 to 4.9

Posterior predictive quantities were generated from the fitted continuous-time structural equation modeling (discrete-time specification in Stan). For each period, the table reports summaries of the posterior predictive distribution of the observed variable.

"Predicted mean/SD" are posterior predictive summaries (mean, SD, and 95% credible interval; 2.5 to 97.5% quantiles across posterior estimates) of the observed variable; "Predicted 2.5th/97.5th percentile" are the lower/upper tails of the posterior predictive distribution of the observed variable.

Study periods: Days 1-29, Days 30-59, and Days ≥60 indicate the elapsed-day strata from the starting point of the experiment; All period pools all observations.

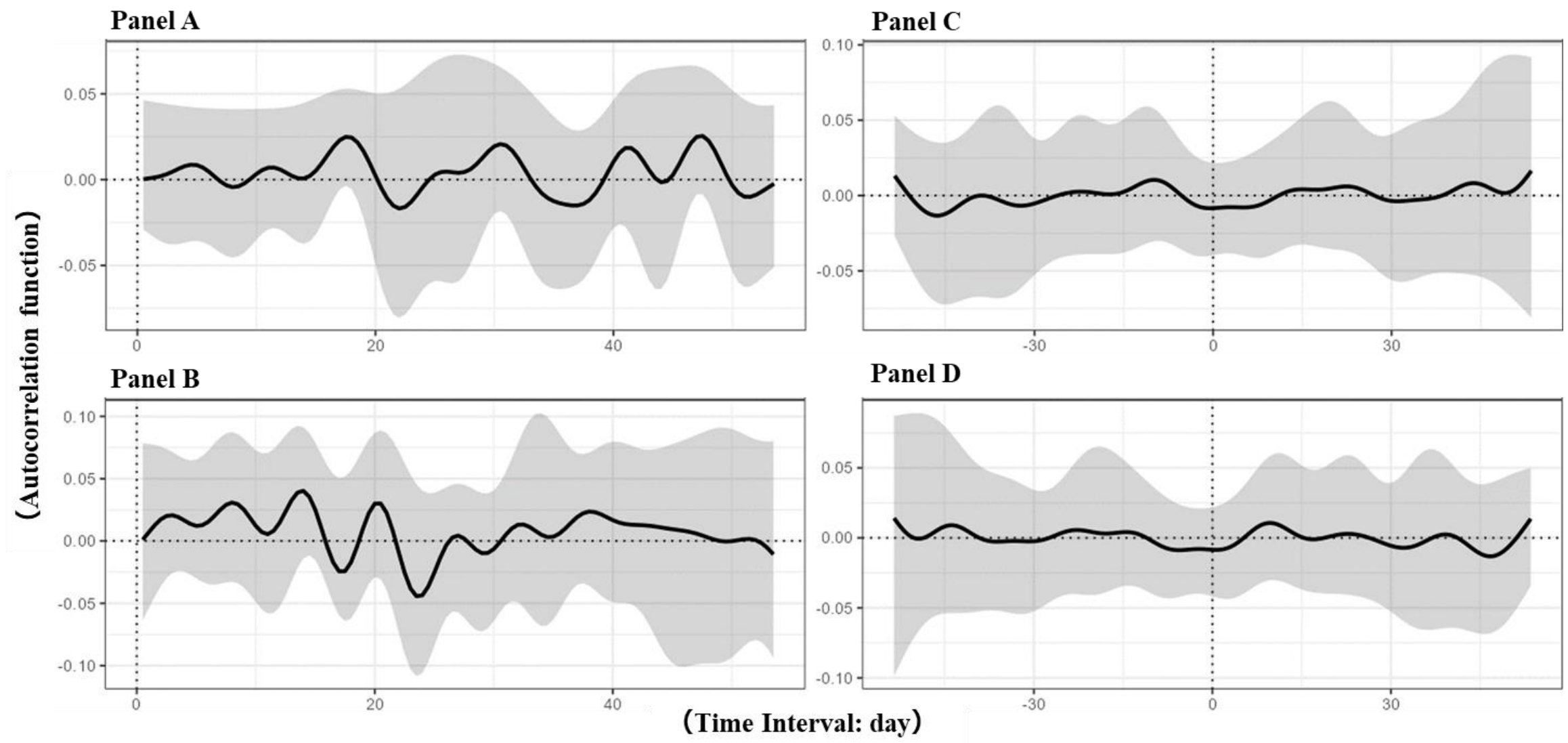

Figure S2: Residual autocorrelation from continuous-time structural equation modeling for explicit and implicit sense of agency

Panel A: Autocorrelation function (ACF) of standardized within-individual drift residuals for local implicit sense of agency (LocalImp-SoA).

Panel B: ACF of standardized within- individual drift residuals for Explicit sense of agency (GenExp-SoA).

Panel C: Cross-correlation function (CCF) between LocalImp-SoA residuals and GenExp-SoA residuals.

Panel D: CCF between GenExp-SoA residuals and LocalImp-SoA residuals.

The solid black line indicates the posterior mean of the (cross-)correlation at each lag, and the gray band represents the 95% Bayesian credible envelope across posterior estimates. The horizontal dotted line indicates zero correlation. The vertical dashed line indicates lag 0. Positive lags are indicative of a correlation with the second series lagged forward in time, while negative lags indicate the first series lagged forward. Lags are expressed in days.

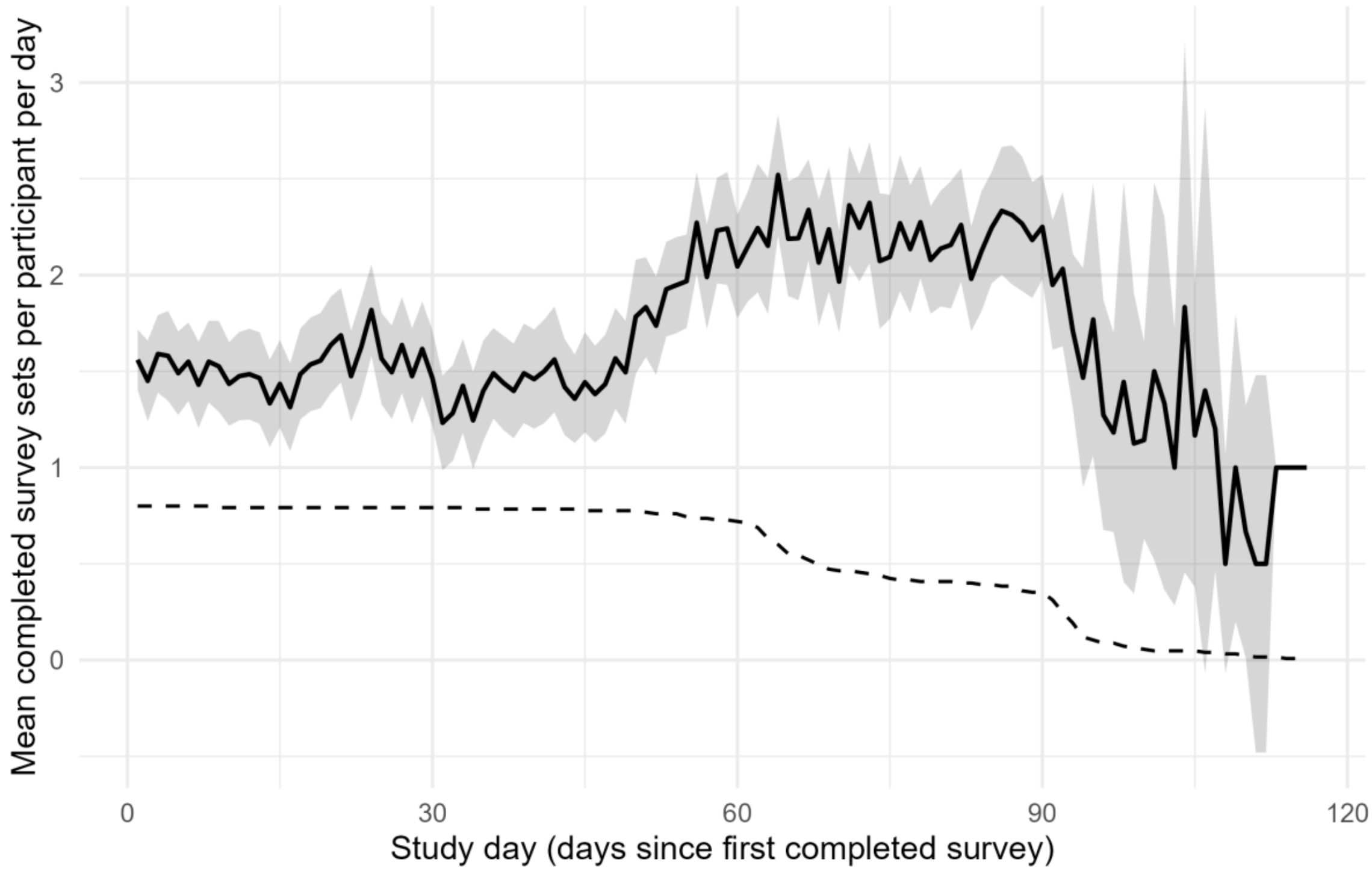

Figure S3. Measurement frequency over time.

The solid line shows the mean number of completed survey sets per participant per day across study days. Study day is defined relative to each participant's first completed survey. The shaded band shows the 95% confidence interval. The dashed line shows the number of participants contributing data on each study day and is scaled to the same vertical axis for visualization. Days without completed surveys were counted as zero. Invitation delivery logs were not available, so the figure summarizes completed surveys and may reflect both nonresponse and technical disruptions. A small number of participants had observation periods from first to last completed day longer than 90 days because of missed days and occasional app reinstallations or resets, which can occur in routine smartphone use. In rare cases, more than three completed survey sets were recorded within a day due to app-level scheduling errors (eg, an additional delivery).
